# Anti-Inflammatory Components from the Root of *Solanum erianthum*

**DOI:** 10.3390/ijms140612581

**Published:** 2013-06-14

**Authors:** Yu-Chang Chen, Hong-Zin Lee, Hsin-Chun Chen, Chi-Luan Wen, Yueh-Hsiung Kuo, Guei-Jane Wang

**Affiliations:** 1 Department of Chinese Pharmaceutical Sciences and Chinese Medicine Resources, College of Pharmacy, China Medical University, Taichung 404, Taiwan; E-Mail: kuoyh@mail.cmu.edu.tw; 2 School of Pharmacy, College of Pharmacy, China Medical University, Taichung 404, Taiwan; E-Mail: hong@mail.cmu.edu.tw; 3 Department of Cosmeceutics, China Medical University, Taichung 404, Taiwan; E-Mail: c0706@mail.cmu.edu.tw; 4 Taiwan Seed Improvement and Propagation Station, Council of Agriculture, Taichung 426, Taiwan; E-Mail: cluwen@tss.gov.tw; 5 Tsuzuki Institute for Traditional Medicine, China Medical University, Taichung 404, Taiwan; 6 Graduate Institute of Clinical Medical Science, China Medical University, Taichung 404, Taiwan; 7 Department of Health and Nutrition Biotechnology, Asia University, Taichung 413, Taiwan

**Keywords:** *Solanum erianthum*, Solanaceae, root, solanerianone, norsesquiterpenoid, sesquiterpenoid, spirovetivene, anti-inflammatory, cytotoxicity

## Abstract

Two new norsesquiterpenoids, solanerianones A and B (**1**–**2**), together with nine known compounds, including four sesquiterpenoids, (−)-solavetivone (**3**), (+)-anhydro-β-rotunol (**4**), solafuranone (**5**), lycifuranone A (**6**); one alkaloid, *N*-*trans*-feruloyltyramine (**7**); one fatty acid, palmitic acid (**8**); one phenylalkanoid, acetovanillone (**9**), and two steroids, β-sitosterol (**10**) and stigmasterol (**11**) were isolated from the *n*-hexane-soluble part of the roots of *Solanum erianthum*. Their structures were elucidated on the basis of physical and spectroscopic data analyses. The anti-inflammatory activity of these isolates was monitored by nitric oxide (NO) production in lipopolysaccharide (LPS)-activated murine macrophage RAW264.7 cells. The cytotoxicity towards human lung squamous carcinoma (CH27), human hepatocellular carcinoma (Hep 3B), human oral squamous carcinoma (HSC-3) and human melanoma (M21) cell lines was also screened by using an MTT assay. Of the compounds tested, **3** exhibited the strongest NO inhibition with the average maximum inhibition (E_max_) at 100 μM and median inhibitory concentration (IC_50_) values of 98.23% ± 0.08% and 65.54 ± 0.18 μM, respectively. None of compounds (**1**–**9**) was found to possess cytotoxic activity against human cancer cell lines at concentrations up to 30 μM.

## 1. Introduction

*Solanum erianthum* D. Don (Solanaceae) is a shrub or small tree with stellate tomentose. It is native to South America and widespread in tropical Asia and Oceania [1]. The leaves are used for the treatment of cancer and malaria in Nigeria [2]. In Taiwan, the leaves are used as maternal tonic and to treat lumbar neuralgia; the stems and roots are used to cure rheumatism and cold; the roots are also used for the treatment of stomachache, abdominal pain, fracture, bruises, and chronic granular leukemia [3,4].

**Figure 1:**
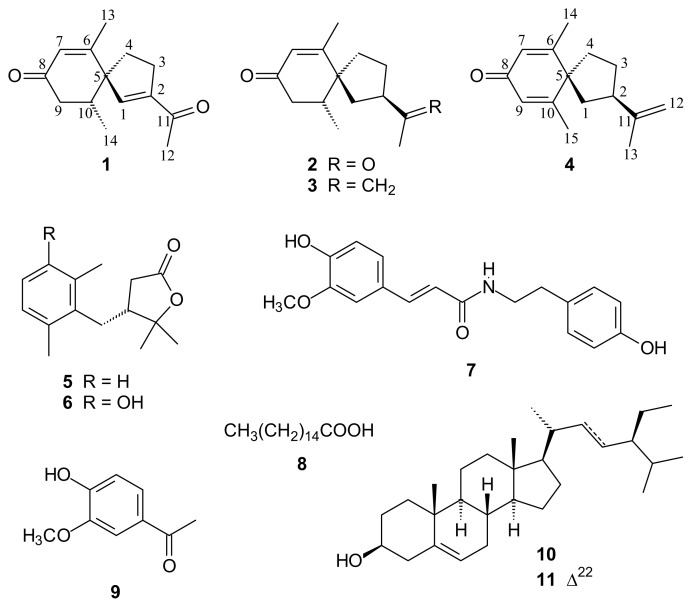
The chemical structures of compounds **1**–**11**.

Flavonoids, steroidal alkaloids, amides and fatty acids were isolated from the leaves of *S. erianthum* [5–8]. The presence of some steroidal alkaloids was detected in the fruits, stem barks, xylem, roots and leaves of this plant by thin-layer chromatography (TLC) [9]. Sesquiterpenes and monoterpenes were the main components in the essential oil from the fruits and leaves of *S. erianthum*, respectively [10,11]. However, there has been no previous report on the chemical constituents of the root of this species. During preliminary screening, the MeOH extract of the root of *S. erianthum* was shown to be able to inhibit NO release without affecting the cellular viability in lipopolysaccharide (LPS)-activated Raw 264.7 cells and display a selective cytotoxic activity against Hep 3B cell line. In the present study, we set out to isolate the active principles from the root extract and to assess the bioactivity of the pure isolates. We now report the isolate of two new solanerianones A and B (**1**–**2**), and nine known compounds, including four sesquiterpenoids, (−)-solavetivone (**3**), (+)-anhydro-β-rotunol (**4**), solafuranone (**5**) and lycifuranone A (**6**); one alkaloid, *N*-*trans*-feruloyltyramine (**7**); one fatty acid, palmitic acid (**8**); one phenylalkanoid, acetovanillone (**9**), and two steroids, β-sitosterol (**10**) and stigmasterol (**11**) from an *n*-hexane-soluble fraction of the root of *S. erianthum* (Figure 1). However, owing to the paucity of plant extracts, some compounds could not be obtained in sufficient quantities for bioassay. Herein, we describe the structure elucidation of these two new compounds, anti-inflammatory activity of compounds **3**–**8** and cytotoxicity evaluation of compounds **1**–**9** against four human cancer cell lines.

## 2. Results and Discussion

### 2.1. Isolation and Structure Elucidation

**Figure 2:**
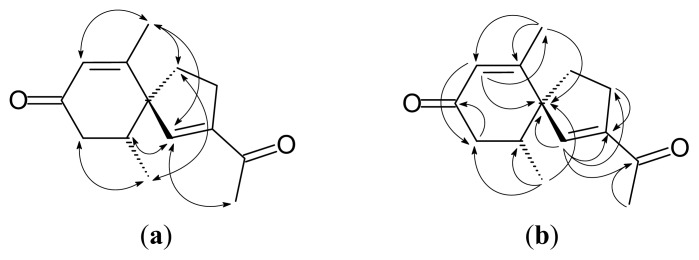
Key NOESY contacts (**a**) and HMBC connectivities (**b**) of compound **1**.

Solanerianone A (**1**) was obtained as colorless oil. Its molecular formula was established as C_14_H_18_O_2_ by EIMS ([M]^+^, *m*/*z* 218, Figure S1) and HREIMS. The presence of an αβ-unsaturated carbonyl with a β-methyl substituent was revealed by the UV (242 nm) [12], IR spectrum data (1665, 1624 cm^−1^), ^1^H-NMR [δ 1.87 (3H, d, *J* = 1.8 Hz, H-13), 5.85 (1H, br s, H-7)] (Table 1, Figures S2 and S3) and ^13^C-NMR [δ 165.5 (C-6), 198.6 (C-8)] (Table 1, Figure S4). ^1^H-NMR (Table 1) suggested a methyl group [δ 2.38 (3H, s, H-12)] adjacent to a carbonyl group [δ 196.4 (C-11)]. The Heteronuclear Multiple Bond Correlation (HMBC) (Figures 2 and S5) showing δ 6.34 (H-1) correlations with δ 147.4 (C-2), and 196.4 (C-11) suggested the presence of another αβ-unsaturated carbonyl moiety. A secondary methyl group [δ 0.92 (3H, d, *J* = 6.0 Hz, H-14)] and a methylene group [δ 2.28 (1H, br d, *J* = 13.2 Hz, H-9), 2.41 (1H, br d, *J* = 13.2 Hz, H-9)] connecting to carbonyl group [δ 198.6 (C-8)] were suggested adjacent to a methine group [δ 2.31 (1H, qt, *J* = 6.0, 1.8 Hz, H-10)]. The HMBC and the Nuclear Overhauser Effect Spectroscopy (NOESY) spectra (Figure 2) suggested C-5 (δ 59.4) is a spirocarbon connecting to C-1, C-4, C-6 and C-10. Cross peaks in the NOESY spectra (Figure 2) distinguished H-10 on the C-1 side from Me-10 on the C-4 side. According to the above data and the specific rotation ( ${\left[\alpha \right]}_{\text{D}}^{23}+27{}^{\circ}$), the structure of **1** was elucidated as (+)-(5*R*,10*R*)-2-(1-ethanone)-6,10-dimethylspiro[4.5]dec-1,6-dien-8-one, which was further confirmed by COSY (Figure S6), NOESY (Figures 2 and S7), ^13^C-NMR (Figure S4), DEPT (Figure S8), HSQC (Figure S9) and HMBC (Figures 2 and S5) experiments.

**Figure 3:**
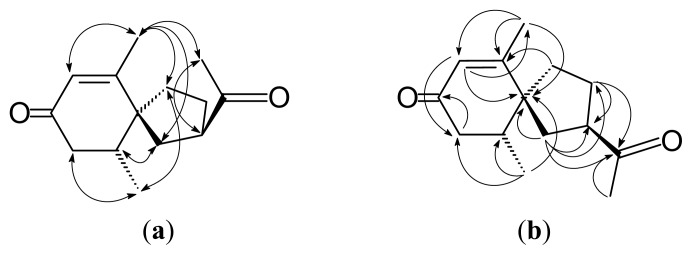
Key NOESY contacts (**a**) and HMBC connectivities (**b**) of compound 2.

Solanerianone B (**2**) was obtained as colorless oil. Its molecular formula was established as C_14_H_20_O_2_ by EIMS ([M]^+^, *m*/*z* 220, Figure S10) and HREIMS. The ^1^H- and ^13^C-NMR spectra (Table 1, Figures S11–S16) of **2** were similar to that of (−)-solavetivone (**3**) [12,13], except that isopropenyl group on **3** was replaced by an acetyl group [δ 2.20 (3H, s, H-12), 3.05 (1H, m, H-2), 51.4 (C-2), 209.6 (C-11)]. Cross peaks (H-10/H-1 and H-14/H-4) in the NOESY spectrum (Figures 3 and S17) distinguished H-10 on the C-1 side from Me-10 on the C-4 side. The NOESY experiment (Figures 3 and S17) showing the cross peak (H-12/H-13) suggested that the acetyl group on C-2 was on the same side of the methyl group on C-6. According to the above data and the specific rotation ( ${\left[\alpha \right]}_{\text{D}}^{24}-86{}^{\circ}$), the structure of **2** was elucidated as (−)-(2*R*,5*S*,10*R*)-2-(1-ethanone)-6,10-dimethylspiro [4.5]dec-6-en-8-one, which was further confirmed by COSY (Figure S18), NOESY (Figures 3 and S17), ^13^C-NMR (Figures S14–S16), DEPT (Figure S19), HSQC (Figure S20) and HMBC (Figures 3 and S21) experiments. Compound **2** have been synthesized [14], but it was first isolated from nature.

The eight known compounds, including (−)-solavetivone (**3**) { ${\left[\alpha \right]}_{\text{D}}^{24}-119{}^{\circ}$ (*c* 1.74, CHCl_3_)}[12,13], (+)-anhydro-β-rotunol (**4**) { ${\left[\alpha \right]}_{\text{D}}^{23}+52{}^{\circ}$ (*c* 0.5, CHCl_3_)} [12,15], solafuranone (**5**) { ${\left[\alpha \right]}_{\text{D}}^{24}+15.0{}^{\circ}$ (*c* 0.09, CHCl_3_)} [16], lycifuranone A (**6**) { ${\left[\alpha \right]}_{\text{D}}^{23}+14{}^{\circ}$ (*c* 0.755, CHCl_3_)} [17], *N*-*trans*-feruloyltyramine (**7**) [18], palmitic acid (**8**) [19], acetovanillone (**9**) [20], and the mixture { ${\left[\alpha \right]}_{\text{D}}^{23}-34.3{}^{\circ}$ (*c* 2.14, CHCl_3_)} of β-sitosterol (**10**) [21] and stigmasterol (**11**) [21] were readily identified by comparison of physical and spectroscopic data (UV, IR, ^1^H-NMR, [α]_D_, and mass spectrometry data) with values found in the literature.

### 2.2. Anti-Inflammatory Activities

**Figure 4:**
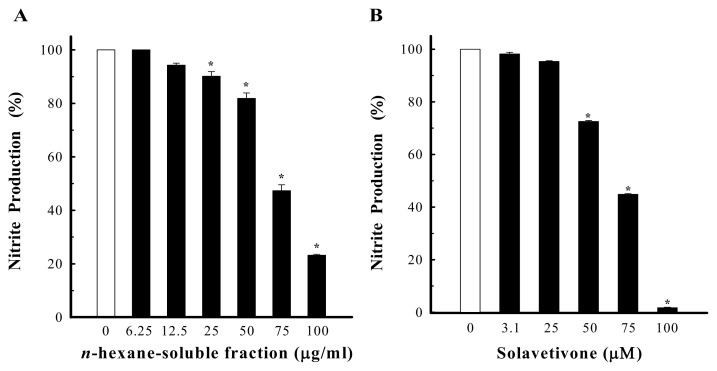
The effect of *n*-hexane-soluble fraction (**A**) and solavetivone (**3**) (**B**) on nitrite production in LPS-activated RAW 264.7 cells. Vehicle representative of 100% is equal to 35.28 ± 0.57 μM of NO produced in the medium per well of cells. *n* = 5–6 in each group. *p < 0.05 when compared with vehicle-treated cells.

NO, overproduced by activated macrophages via inducible NO synthase (iNOS), is suggested to be a significant pathogenic factor in various inflammatory tissue injuries. In order to elucidate the anti-inflammatory action of the root of *S. erianthum*, the present study was designed to isolate its active constituents and examine their effects on NO production, detected as nitrite in the culture medium, induced by LPS through iNOS expression in RAW264.7 cells, to reflect the degree of anti-inflammatory activity. By the guidance of the bioassay, the *n*-hexane-soluble fraction was isolated to exhibit a significant bioactivity without affecting the cellular viability at a concentration of 100 μg/mL; the inhibition being 76.78% ± 0.34% and with an IC_50_ value of 72.80 ± 1.50 μg/mL (Figure 4A). This finding prompted us to investigate the active principles from this fraction, and led to the isolation and identification of two novel norsesquiterpenoids along with eight known compounds. The effects of **3**–**8** on the inhibition of NO production in LPS-activated RAW264.7 cells were evaluated. E_max_ (%) and IC_50_ (μM) values of iNOS inhibitory activity were obtained at the concentration range of 3.0 to 100 μM. Results are shown in Table 2. Among the isolates, solavetivone (**3**) which was the major compound in *n*-hexane-soluble fraction exhibited a significant activity, with the E_max_ and IC_50_ values of 98.23% ± 0.08% and 65.54 ± 0.18 μM, respectively. As shown in Figure 4B, it inhibited LPS-induced NO production in a concentration-dependent manner. (+)-Anhydro-β-rotunol (**4**) was similar to **3** belonging to spirovetivene sesquiterpenoid, but it showed a mild effect. It suggested that the stereochemistry of the double bond between C-9 and C-10 of **4** decreased the iNOS inhibitory activity. Solafuranone (**5**) and lycifuranone A (**6**) contain the same carbon skeleton which may be biologically converted from **3**[16], and they seem not to be linked to NO production at the tested concentrations, although it did not affect cell viability. *N*-*trans*-Feruloyltyramine (**7**) exhibited moderate iNOS inhibitory activity, with E_max_ value of 33.33% ± 1.69%. The iNOS inhibitory activity of palmitic acid (**8**) showed weak effect, with E_max_ value of 13.22% ± 1.11%. Under the same conditions, the maximum inhibitory effects of positive controls aminoguanidine (a selective iNOS inhibitor) and *N^ω^*-nitro-l-arginine (a nonselective iNOS inhibitor) were 80.97% ± 0.63% and 42.19% ± 0.94%, respectively.

### 2.3. Cytotoxicity Assay

The isolates were evaluated for their cytotoxic activities against four human cancer cell lines: CH27 (human lung squamous carcinoma), Hep 3B (human hepatocellular carcinoma), HSC-3 (human oral squamous carcinoma), M21 (human melanoma) using the MTT method. The results showed that they were inactive at 30 μM (data not shown). The known (−)-solavetivone (**3**) with cytotoxicity against OVCAR-3 cancer cell line (IC_50_: 0.1 mM)[16] and the known *N*-*trans*-feruloyltyramine (**7**) with cytotoxicity against P-388 and HL-60 cancer cell lines with the medium effect dose (ED_50_) values of 7.97 μg/mL and 2.90 μg/mL, respectively] [22] were reported.

## 3. Experimental Section

### 3.1. General

Silica gel *60 F_254_* precoated plates (*Merck*). Column chromatography (CC): silica gel (*SiliCycle* 60–200 μm, 40–63 μm). Medium performance liquid chromatography (MPLC): silica gel (*SiliCycle* 15–35 μm); pump: *FIM LAB PUMP: QSY*. M.p.: *Yanaco* micro-melting-point apparatus; uncorrected. Optical rotation: *Jasco-DIP-370* polarimeter; in CHCl_3_. UV Spectra: *Jasco-UV-240* spectrophotometer; λ_max_ (log ɛ) in nm. IR Spectra: *Shimadzu-IRPrestige-21* FT-IR spectrophotometer; ν in cm^−1. 1^H-, ^13^C-, and 2D-NMR Spectra: *Varian-Inova-600* and *Bruker-AVA-400* spectrometers; δ in ppm rel. to SiMe_4_, *J* in Hz. EI-MS and HR-EI-MS: *Finnigan/Thermo Quest MAT 95XL* mass spectrometer; *m*/*z* (rel. %). All reagents were purchased from Sigma-Aldrich Chemical Co. (St. Louis, MO, USA) unless otherwise stated.

### 3.2. Plant Material

The roots of *S. erianthum* were collected from Tainan, Taiwan, in July 2009 and identified by Dr. Yu-Chang Chen. A voucher specimen (YCC 0971) has been deposited in the Herbarium of the College of Pharmacy, China Medical University, Taichung, Taiwan.

### 3.3. Extraction and Isolation

Dried roots (12.8 kg) of *S. erianthum* were sliced and extracted with cold MeOH three times. After removal of the solvent under vacuum, the extract was partitioned into an *n*-hexane-soluble fraction (fraction A, 47.8 g), a EtOAc-soluble fraction (fraction B, 54.0 g), an *n*-BuOH-soluble fraction (fraction C, 335.0 g), and a water-soluble fraction (fraction D, 431.8 g).

Fraction A (47.5 g) was chromatographed using silica gel (1.5 kg), eluting initially with CH_2_Cl_2_ and gradually enriching with acetone to give 25 fractions. Fraction A-4 (5.19 g, CH_2_Cl_2_, 100%) was washed with *n*-hexane to obtain white crystal (552.6 mg). The crystal (552.6 mg) was chromatographed on silica gel (30 g), eluting with *n*-hexane-CHCl_3_ (1:2) to give 2 fractions. Fraction A-4-1 (457.6 mg) was further purified by MPLC (*n*-hexane-CHCl_3_, 1:1) to obtain **5** (98.8 mg). Fraction A-6 (3.99 g, CH_2_Cl_2_-acetone, 99:1) was chromatographed on silica gel (120 g), eluting with *n*-hexane and gradually increasing the polarity with acetone to give 11 fractions. Fraction A-6-3 (670.8 mg, *n*-hexane-acetone, 98:2) was further purified by MPLC (CH_2_Cl_2_, 100%) to afford **8** (261.7 mg). Fraction A-7 (5.98 g, CH_2_Cl_2_-acetone, 99:1) was chromatographed on silica gel (180 g), eluting with *n*-hexane and gradually increasing the polarity with acetone to obtain **3** (1.65 g) and a mixture (336.5 mg) of **10** and **11**. Fraction A-8 (817.0 mg, CH_2_Cl_2_-acetone, 99:1) was chromatographed on silica gel (25 g), eluting with CH_2_Cl_2_ and gradually increasing the polarity with EtOAc to give 5 fractions. Fractions A-8-2 (111.0 mg, CH_2_Cl_2_-EtOAc, 99:1) and A-8-3 (454.0 mg, CH_2_Cl_2_-EtOAc, 99:1) were further purified by MPLC (*n*-hexane-acetone, 8:1) to afford **3** (16.1 mg) and **9** (1.5 mg), respectively. Fraction A-12 (584.2 mg, CH_2_Cl_2_-acetone, 97:3) was chromatographed on silica gel (30 g), eluting with CHCl_3_ and gradually increasing the polarity with MeOH to obtain 12 fractions. Fraction A-12-2 (82.5 mg, CHCl_3_-MeOH, 90:1) was submitted to MPLC (*n*-hexane-EtOAc, 10:1) to give 13 fractions. Fraction A-12-2-2 (18.8 mg) was further purified by TLC (*n*-hexane-EtOAc, 2:1) to afford **4** (21.3 mg). Fraction A-12-3 (80.1 mg, CHCl_3_-MeOH, 90:1) was subjected to MPLC (*n*-hexane-EtOAc, 5:1) to give 11 fractions. Fraction A-12-3-8 (5.7 mg) was further purified by TLC (*n*-hexane-EtOAc, 3:2) to afford **1** (1.6 mg). Fraction A-12-5 (123.3 mg, CHCl_3_-MeOH, 90:1) was submitted to MPLC (*n*-hexane-acetone, 5:1) to give 8 fractions. Fraction A-12-5-6 (18.8 mg) was further purified by TLC (CH_2_Cl_2_-EtOAc, 4:1) to obtain **6** (17.8 mg). Fraction A-13 (1.89 g, CH_2_Cl_2_-acetone, 95:5) was chromatographed on silica gel (70 g), eluting with *n*-hexane-CHCl_3_ (1:3) and gradually increasing the polarity with CHCl_3_ to give 11 fractions. Fraction A-13-3 (400.0 mg, *n*-hexane-CHCl_3_, 1:3) was subjected to MPLC (*n*-hexane-EtOAc, 2:1) to obtain 10 fractions. Fraction A-13-3-7 (25.7 mg) was further purified by MPLC (CHCl_3_-EtOAc, 20:1) to afford **2** (1.3 mg). Fraction A-18 (436.5 mg, CH_2_Cl_2_-acetone, 85:15) was subjected to MPLC (*n*-hexane-EtOAc, 1:1) to obtain 10 fractions. Fraction A-18-8 (36.4 mg) was submitted to MPLC (*n*-hexane-acetone, 3:2) and then purified by TLC (*n*-hexane-acetone, 1:1) to give **7** (5.9 mg).

### 3.4. Solanerianone A (1)

Colorless oil; ${\left[\alpha \right]}_{\text{D}}^{23}+27{}^{\circ}$ (*c* 0.09, CHCl_3_); UV (MeOH) λ_max_ (log ɛ): 242 (4.15) nm; IR (neat) ν_max_: 2959, 2930, 1665 (C=O), 1624, 1429, 1375 cm^−1^; EIMS: *m/z* (rel. int.): 218 (M^+^, 20), 177 (14), 176 (100), 161 (17), 133 (41), 105 (11); HREIMS: 218.1302 (C_14_H_18_O_2_^+^, calc. 218.1304); ^1^H- and ^13^C-NMR: see Table 1.

### 3.5. Solanerianone B (2)

Colorless oil; ${\left[\alpha \right]}_{\text{D}}^{24}-86{}^{\circ}$ (*c* 0.065, CHCl_3_); UV (MeOH) λ_max_ (log ɛ): 241 (4.03) nm; IR (neat) ν_max_: 2961, 2889, 1705 (C=O), 1665 (C=O), 1431, 1369 cm^−1^; EIMS: *m/z* (rel. int.): 220 (M^+^, 26), 205 ([M-CH_3_]^+^, 13), 178 (42), 177 (27), 163 (12), 150 (14), 137 (40), 135 (100), 121 (17), 108 (26), 107 (27), 91 (33); HREIMS: 220.1470 (C_14_H_20_O_2_^+^, calc. 220.1463); ^1^H- and ^13^C-NMR: see Table 1.

### 3.6. Anti-Inflammatory Activity Assay

#### 3.6.1. Cell Culture

RAW 264.7 cells, a transformed murine macrophage cell line, obtained from the Bioresource Collection and Research Center (Hsinchu, Taiwan), were maintained by twice-weekly passage in DMEM supplemented with 10% fetal calf serum (FCS; HyClone Laboratories, Logan, UT, USA) and penicillin-streptomycin.

#### 3.6.2. Evaluation of NO Product by Nitrite Measurement

Nitrite measurement was based on our previously published technique [23]. Cell aliquots (5 × 10^5^ cells/mL) were grown to confluence on 24-well plates for 24 h. The medium was changed to serum-free DMEM for another 4 h to render the attached cells quiescent. To assess the effects on LPS-induced NO production, compounds, two positive controls aminoguanidine (a selective iNOS inhibitor; 100 μM) and *N^ω^*-nitro-l-arginine (a non-selective NOS inhibitor; 100 μM) or vehicle (0.1% DMSO) were added in the presence of LPS (200 ng/mL) to the cells for another 24 h. The culture supernatant was subsequently collected for nitrite assay as a reflection of NO production [24]. Briefly, an aliquot of supernatant was mixed with an equal volume of Griess reagent (prepared by adding 1 part 0.1% napthylethylenediamine dihydrochloride to 1 part 1% sulfanilamide in 5% phosphoric acid) and incubated at room temperature for 10 min. The absorbance at 550 nm was measured by a microplate spectrophotometer (Bio-Tek Instrument, Inc., Winooski, VT, USA). Fresh medium was used as the blank. The nitrite concentration was determined by reference to a standard curve, using sodium nitrite diluted in the stock culture medium. Results are expressed as percentage of inhibition calculated *versus* vehicle plus LPS-treated cells.

#### 3.6.3. Cell Viability Assay

A redox indicator, alamarBlue, was used to measure the cytotoxicity as has been described previously [25]. After culture supernatant was removed for nitrite measurement as described above, a solution of 10% alamarBlue in DMEM was added to each well containing RAW 264.7 cells. The plates were incubated at 37 °C in a humidified 5% CO_2_ for 1 h. Following incubation, the absorbance of the alamarBlue was read spectrophotometrically at dual wavelengths of 570 and 600 nm against the blank prepared from cell-free wells. The absorbance in cultures treated with LPS plus vehicle was regarded as indicating 100% cell viability.

### 3.7. Cytotoxicity Assay

#### 3.7.1. Cell Culture

The M21 cells were grown in monolayer culture in RPMI medium 1640 (Invitrogen, Carlsbad, CA, USA) containing 10% fetal calf serum (FCS; HyClone Laboratories, Logan, UT, USA), 100 U/mL penicillin and 100 μg/mL streptomycin (Invitrogen) at 37 °C in a humidified atmosphere comprised of 95% air and 5% CO_2_. CH27 and Hep3B cells were cultured in Dulbecco’s modified Eagle’s medium (DMEM; Invitrogen) containing 5% FCS and penicillin-streptomycin and 2 mM glutamine (Merck, Darmstadt, Germany). HSC-3 cells were cultured in DMEM/F12 (1:1) with L-glutamine and HEPES (HyClone) containing 5% FCS and penicillin-streptomycin. When cells were treated with test compounds, the culture medium containing 1% FCS was used.

#### 3.7.2. Mitochondrial Reductase Activity

Cells were seeded at a density of 5 × 10^4^ cells per well onto 12-well plates 48 h before being treated with test compounds. The cells were incubated with 30 μM of test compounds for 24 h. The control cultures were treated with 0.1% dimethyl sulfoxide (DMSO). After treatment, the cells were washed with phosphate-buffered saline (PBS). Cellular mitochondrial reductase activity of live cells was determined by measuring the reduction of 3-(4,5-dimethylthiazol-2-yl)-2,5-diphenyltetrazolium bromide (MTT). At each end point, the treatment medium was replaced with fresh serum-free medium containing 2.4 × 10^−4^ M MTT at pH 7.4. Cells were incubated with MTT medium for 1 h at 37 °C. After solubilization in DMSO, absorbance was measured at 550 nm.

### 3.8. Statistical Analysis

For each experimental series, data are given as mean ± S.E., with *n* representing the number of independently performed experiments. Comparisons of the concentration and treatment effects were conducted with ANOVA, followed by post hoc comparisons by Newman Keuls test as appropriate. The average IC_50_ value was determined by data fitting with GraFit (Erithacus Software, Surrey, UK). A *p* value of less than 0.05 was considered to indicate a statistically significant difference.

## 4. Conclusions

Solanerianones A and B (**1**–**2**) were new norsesquiterpenoids which contain a carbon skeleton similar to (−)-solavetivone (**3**) except that isopropenyl group at C-2 was replaced by an acetyl group. Compounds **3**–**7** and **9** were first isolated from *S. erianthum*. The spirovetivene compound **3**, the major active constituent in *n*-hexane-soluble fraction of the root of this plant, significantly inhibited NO production of RAW264.7 cells without any cytotoxicity. It was reported to have fungitoxicity [12], antimicrobial activity [26] and weak cytotoxicity [16], but its anti-inflammatory activity was studied first. Other spirovetivene compounds were reported to show spasmolytic activity [27] and an induction effect on brain-derived neurotrophic factor mRNA expression [28]. On the other hand, compound **7** exhibited moderate iNOS inhibitory activity. These findings suggest that naturally occurring iNOS inhibitors **3** and **7** may provide a rationale for the potential anti-inflammatory effect of *S. erianthum*. The yields of compounds **1** and **2** were too low to study the anti-inflammatory activities. Compound **2** will be prepared from **3** by oxidative cleavage of the methylene group following the Tamariz technique [29] in the future. However, it is difficult to prepare compound **1** from **3**. Therefore compound **1** should be prepared by total synthesis unless a proper precursor is obtained.

## Supplementary Information



## Figures and Tables

**Table 1:** ^1^H-(600 MHz) and ^13^C-NMR (150 MHz) data (CDCl_3_) of compounds **1** and **2**. Chemical shifts δ in ppm relative to tetramethylsilant (TMS), *J* in Hz.

Position	1		2	
		
	δ_H_	δ_C_	δ_H_	δ_C_
1	6.34 (t, *J* = 1.8, 1H)	145.7	1.96 (m, 1H)	37.5
			2.13 (m, 1H)	

2	-	147.4	3.05 (m, 1H)	51.4

3	2.60 (m, 1H)	31.2	1.91 (m, 1H)	29.4
	2.72 (m, 1H)		2.01 (m, 1H)	

4	1.79 (ddd, *J* = 14.4, 9.6, 7.2, 1H)	27.2	1.75 (m, 1H)	33.2
	2.22 (ddd, *J* = 14.4, 9.6, 4.8, 1H)		1.89 (m, 1H)	

5	-	59.4	-	50.8

6	-	165.5	-	165.1

7	5.85 (br s, 1H)	126.1	5.80 (br s, 1H)	126.4

8	-	198.6	-	198.6

9	2.28 (br d, *J* = 13.2, 1H)	41.9	2.23 (dd, *J* = 16.8, 4.8, 1H)	42.6
	2.41 (br d, *J* = 13.2, 1H)		2.64 (dd, *J* = 16.8, 4.8, 1H)	

10	2.31 (qt, *J* = 6.0, 1.8, 1H)	37.2	2.11 (m, 1H)	37.7

11	-	196.4	-	209.6

12	2.38 (s, 3H)	27.0	2.20 (s, 3H)	29.4

13	1.87 (d, *J* = 1.8, 3H)	21.4	1.93 (d, *J* = 1.2, 3H)	21.0

14	0.92 (d, *J* = 6.0, 3H)	15.9	0.99 (d, *J* = 7.2, 3H)	15.9

**Table 2:** Mean E_max_ and IC_50_ of compounds **3**–**8** on nitrite production induced by LPS in RAW 264.7 cells.

Compound	E_max_ (%)	IC_50_ (μM)
(−)-Solavetivone (**3**)	98.23 ± 0.08	65.54 ± 0.18
(+)-Anhydro-β-rotunol (**4**)	8.77 ± 1.24	>100
Solafuranone (**5**)	0 ± 0	>100
Lycifuranone A (**6**)	0 ± 0	>100
*N*-*trans*-Feruloyltyramine (**7**)	33.33 ± 1.69	>100
Palmitic acid (**8**)	13.22 ± 1.11	>100
Positive control aminoguanidine (a selective iNOS inhibitor)	80.97 ± 0.63	22.28 ± 0.47
*N^ω^*-nitro-l-arginine (a nonselective iNOS inhibitor)	42.19 ± 0.94	147.33 ± 7.61

E_max_ indicates mean maximum inhibitory effect, at concentration of 100 μM, expressed as a percentage inhibition of nitrite production induced by LPS (200 ng/mL) in the presence of vehicle; IC_50_, mean concentration producing 50% E_max_. *n* = 4–6 in each group.
