# Supplementary material for: Anti-Inflammatory Components from the Root of Solanum erianthum
Source: Int J Mol Sci. 2013 Jun 14;14(6):12581–92. doi: 10.3390/ijms140612581 (PMC3709801; doi:10.3390/ijms140612581)

Supporting Information

Figure S1. EIMS spectrum of solanerianone A (**1**).

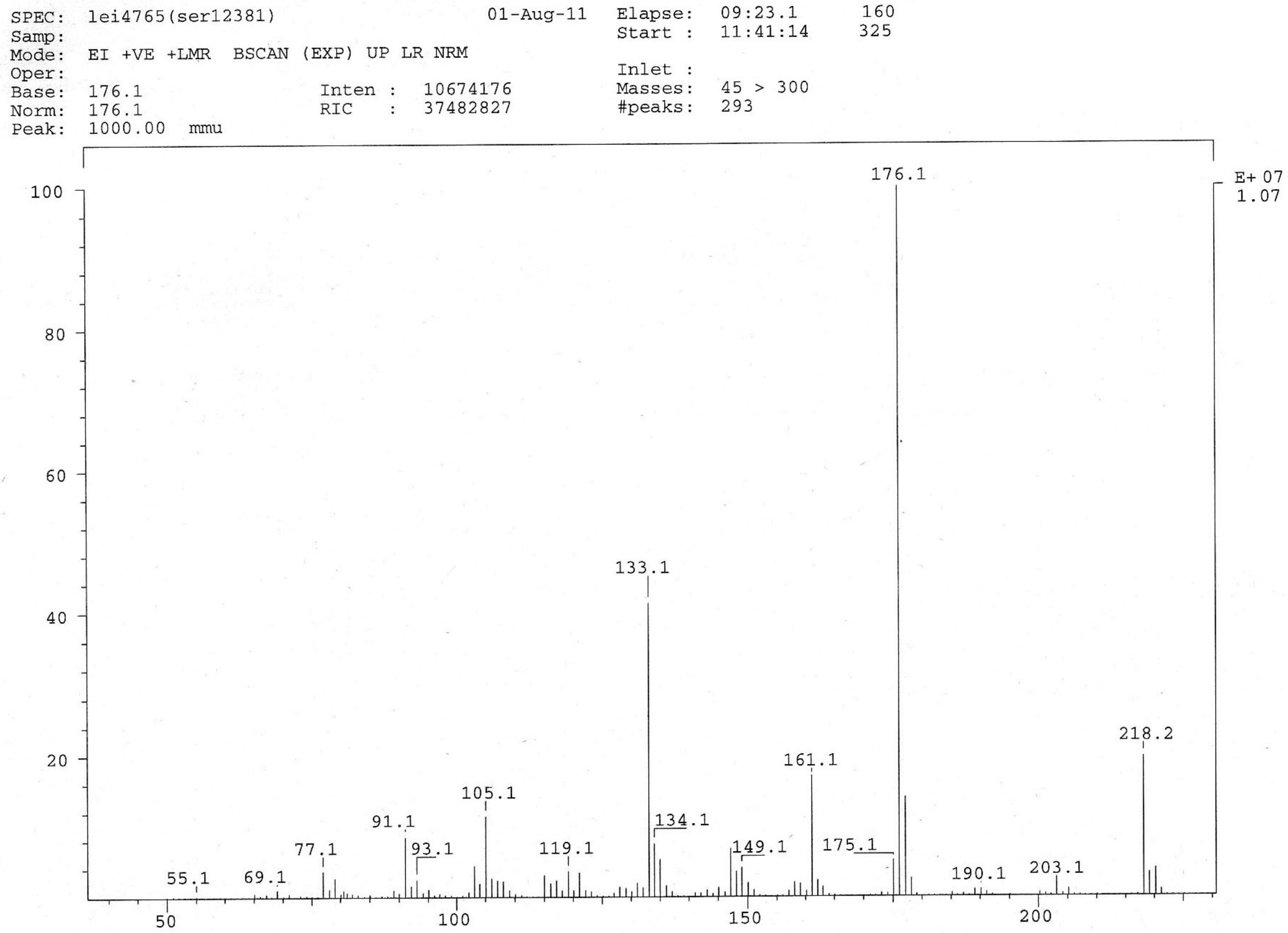

Figure S2. ^{1}H-NMR spectrum of solanerianone A (**1**; 600 MHz, $CDCl_3$).

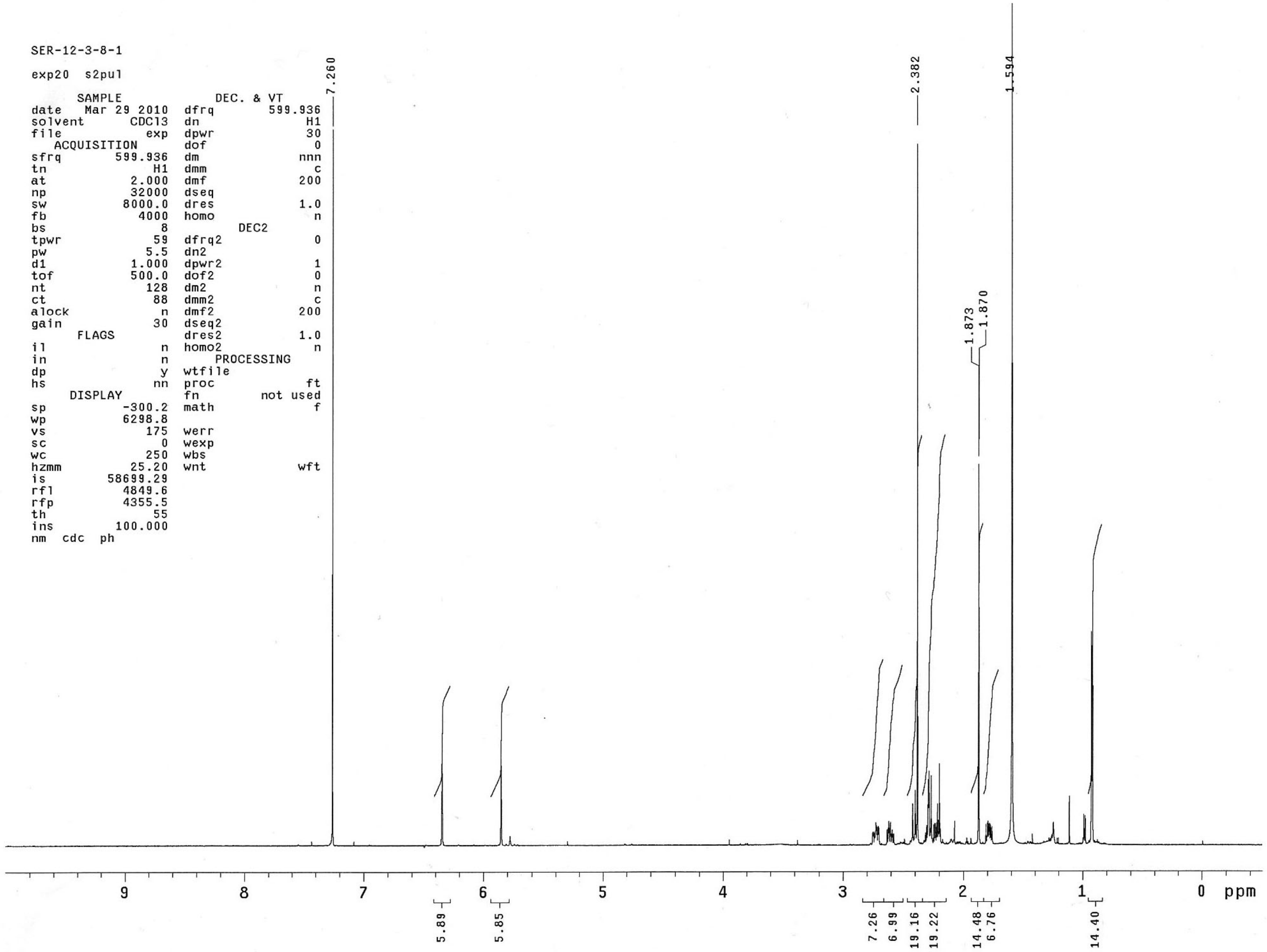

Figure S3. The magnified ^{1}H-NMR spectrum of solanerianone A (**1**; 600 MHz, $CDCl_3$).

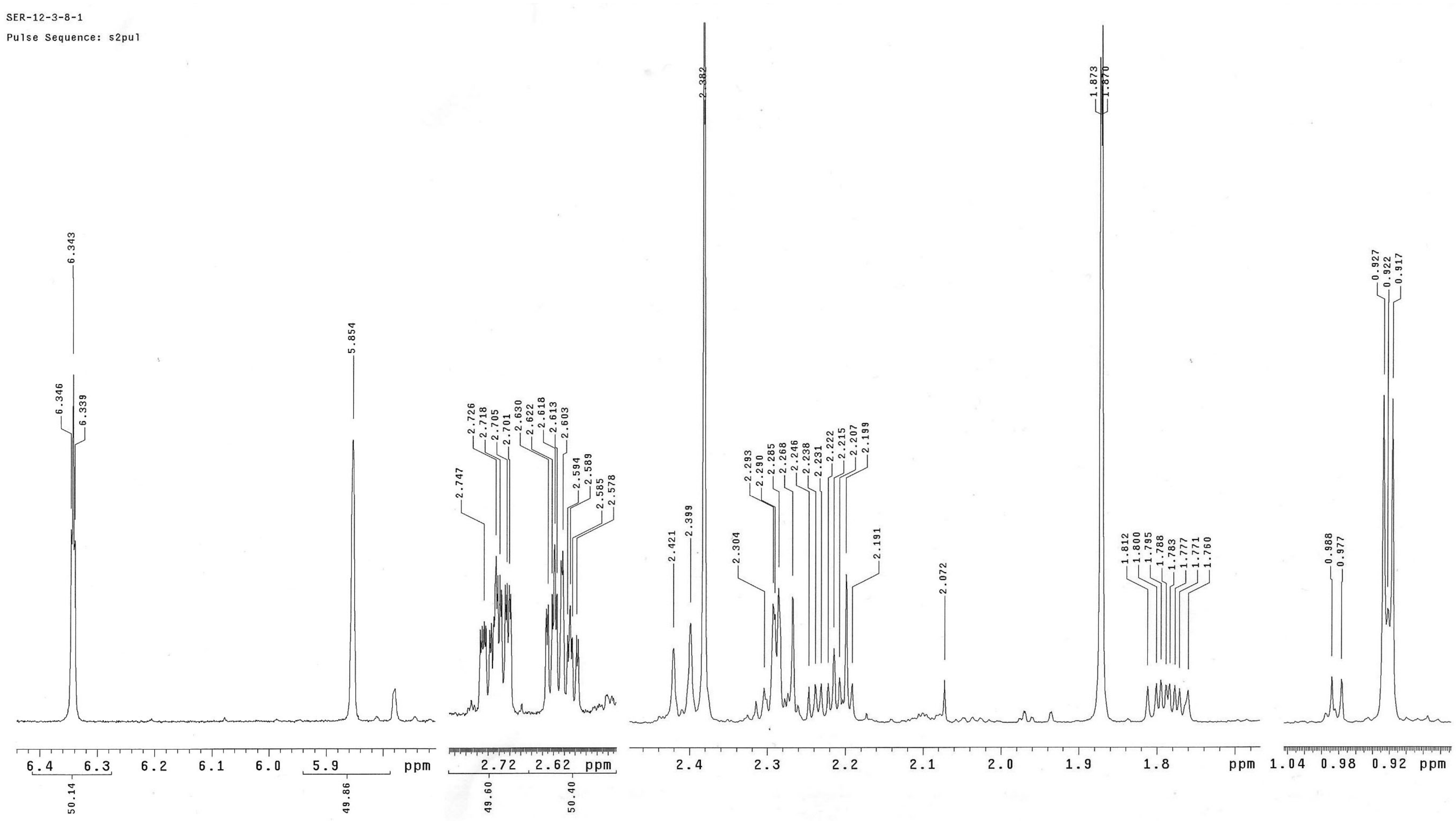

Figure S4. ^{13}C-NMR spectrum of solanerianone A (**1**; 150 MHz, $CDCl_3$).

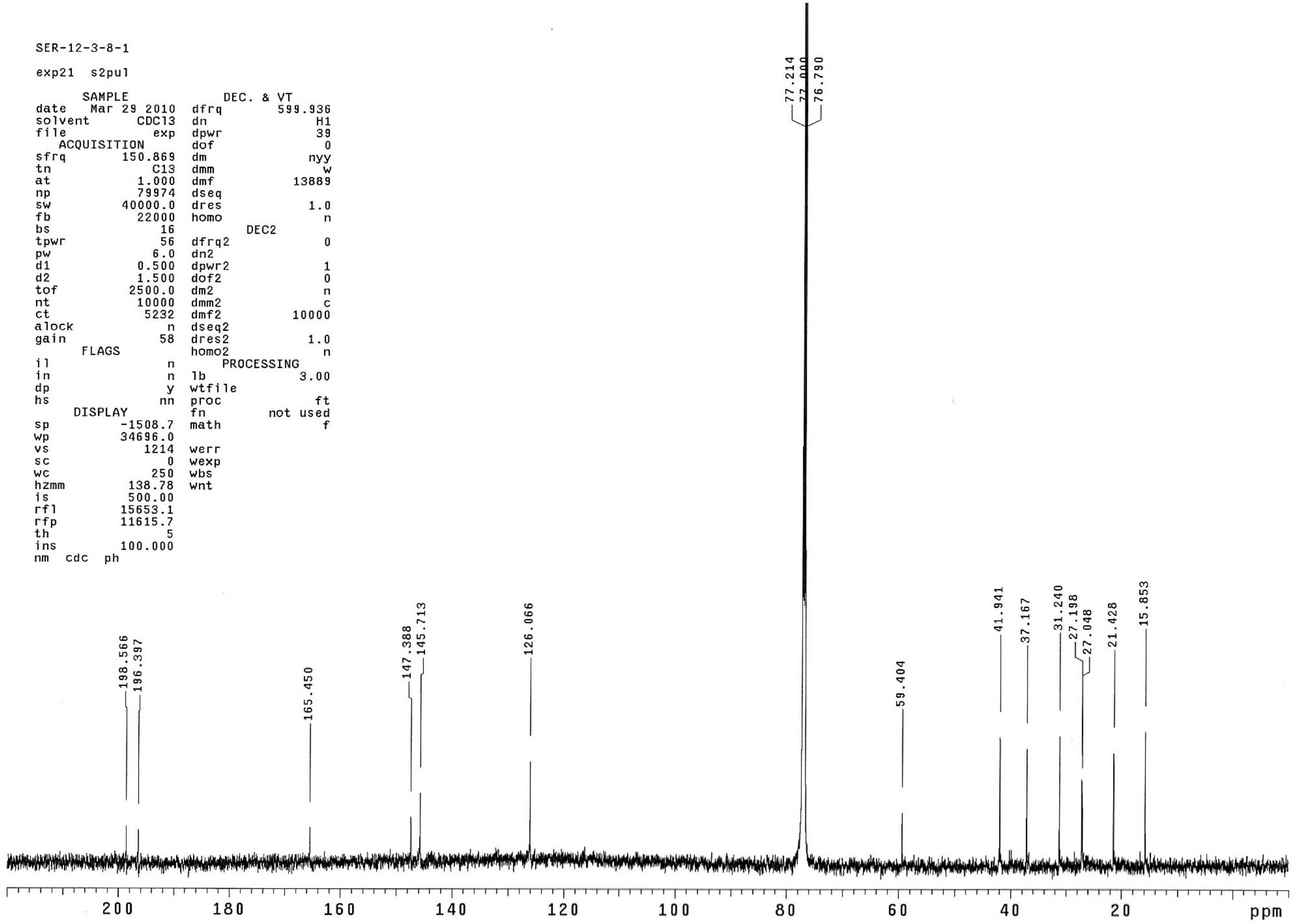

Figure S5. HMBC spectrum of solanerianone A (**1**).

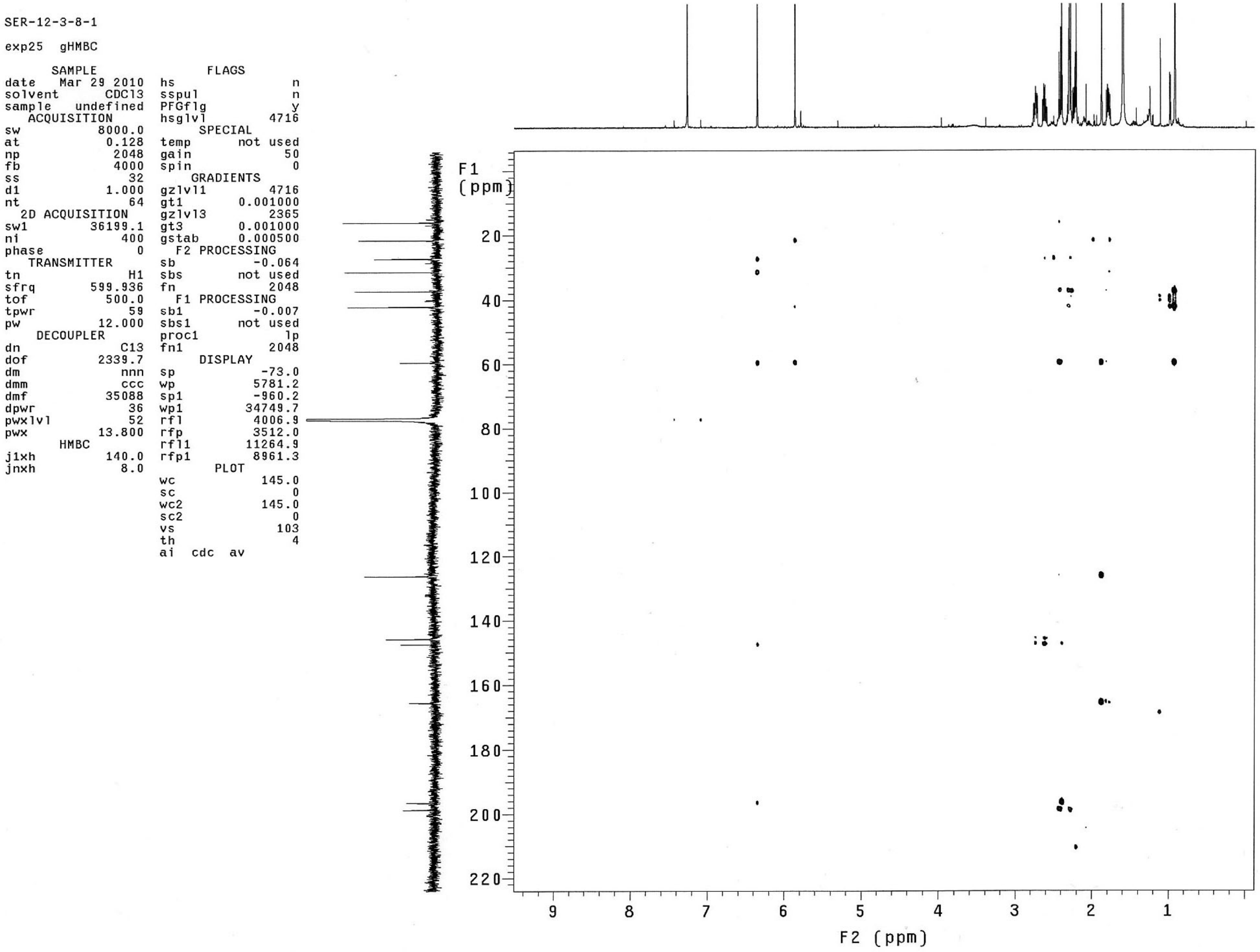

Figure S6. COSY spectrum of solanerianone A (**1**).

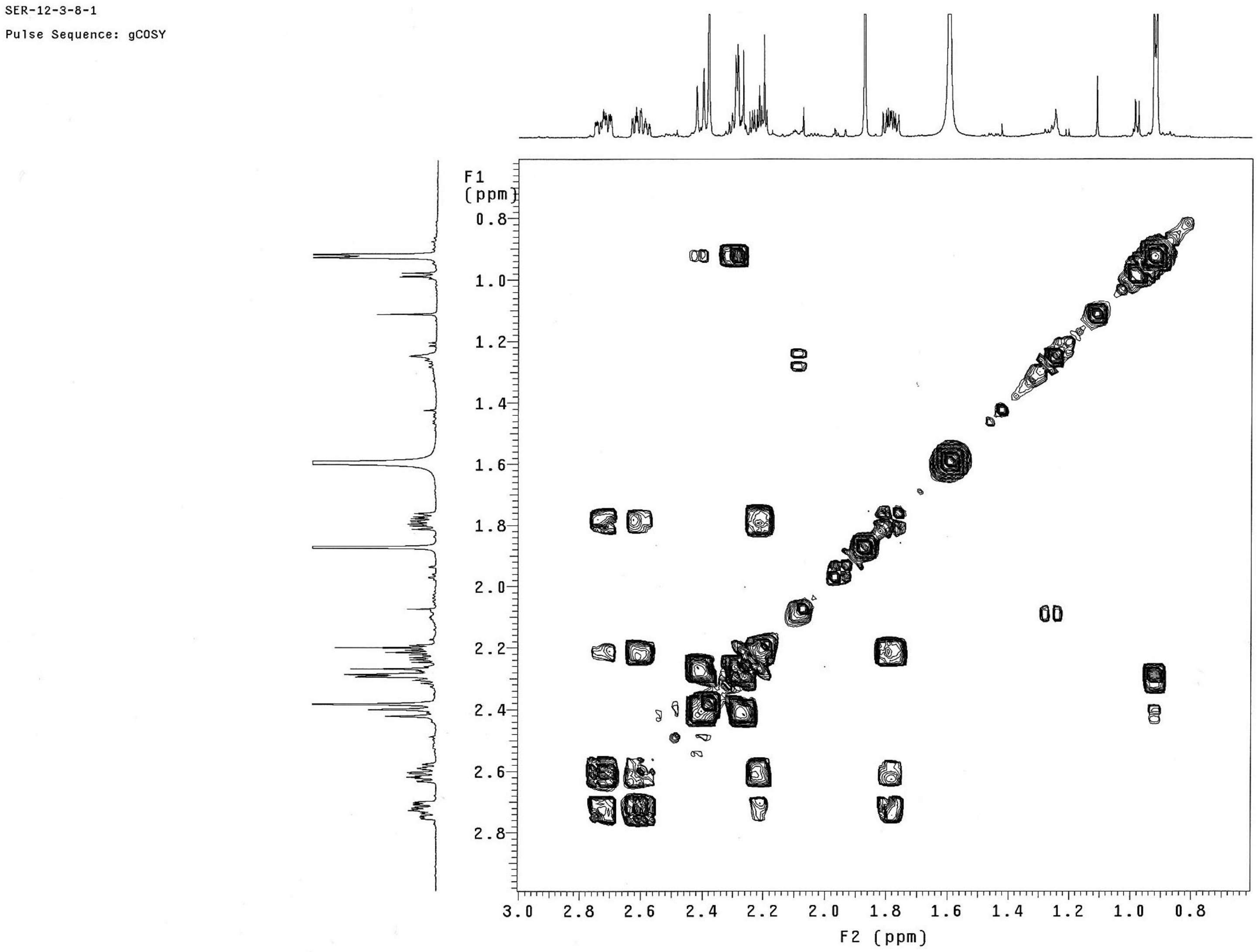

Figure S7. NOESY spectrum of solanerianone A (**1**).

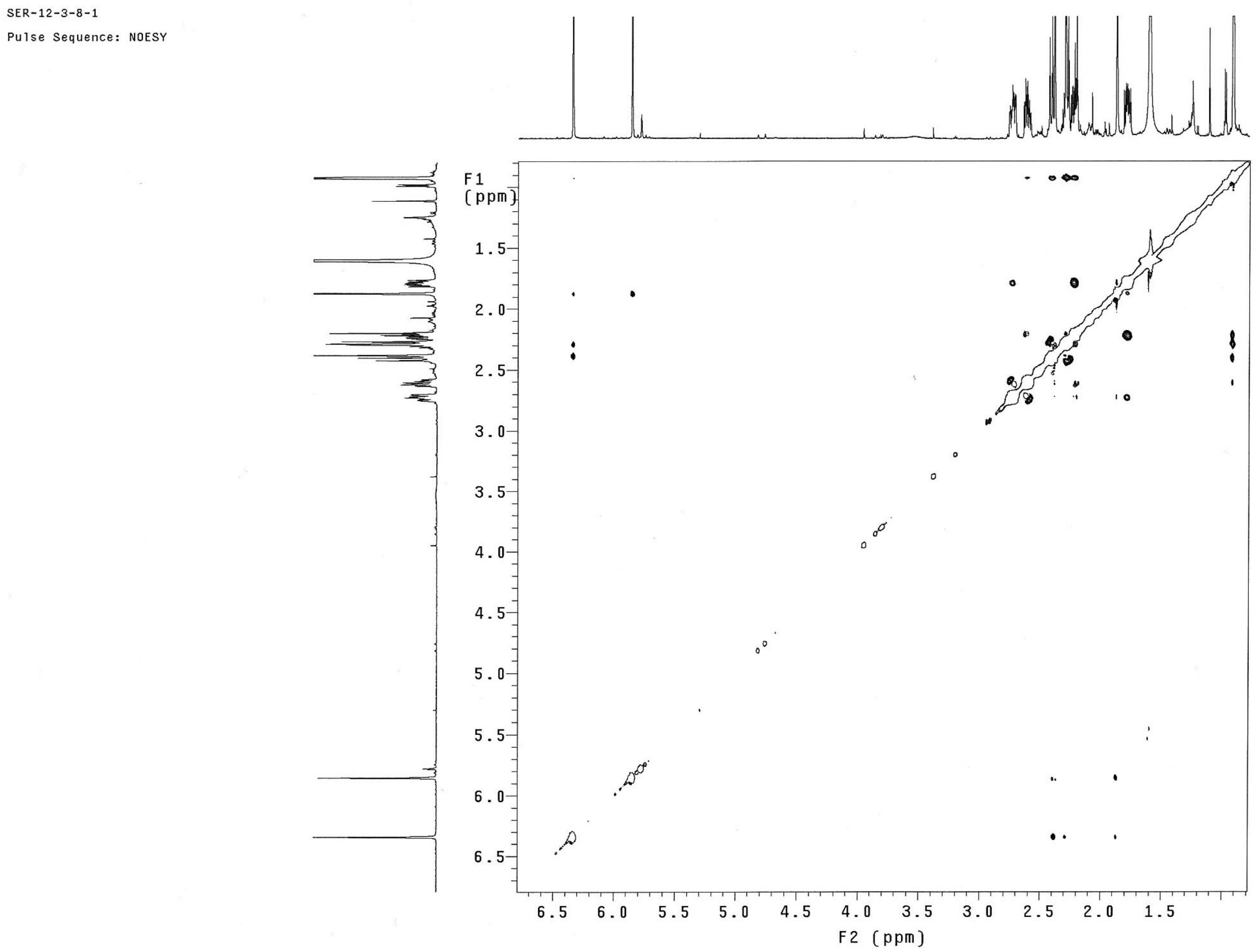

Figure S8. DEPT spectrum of solanerianone A (**1**).

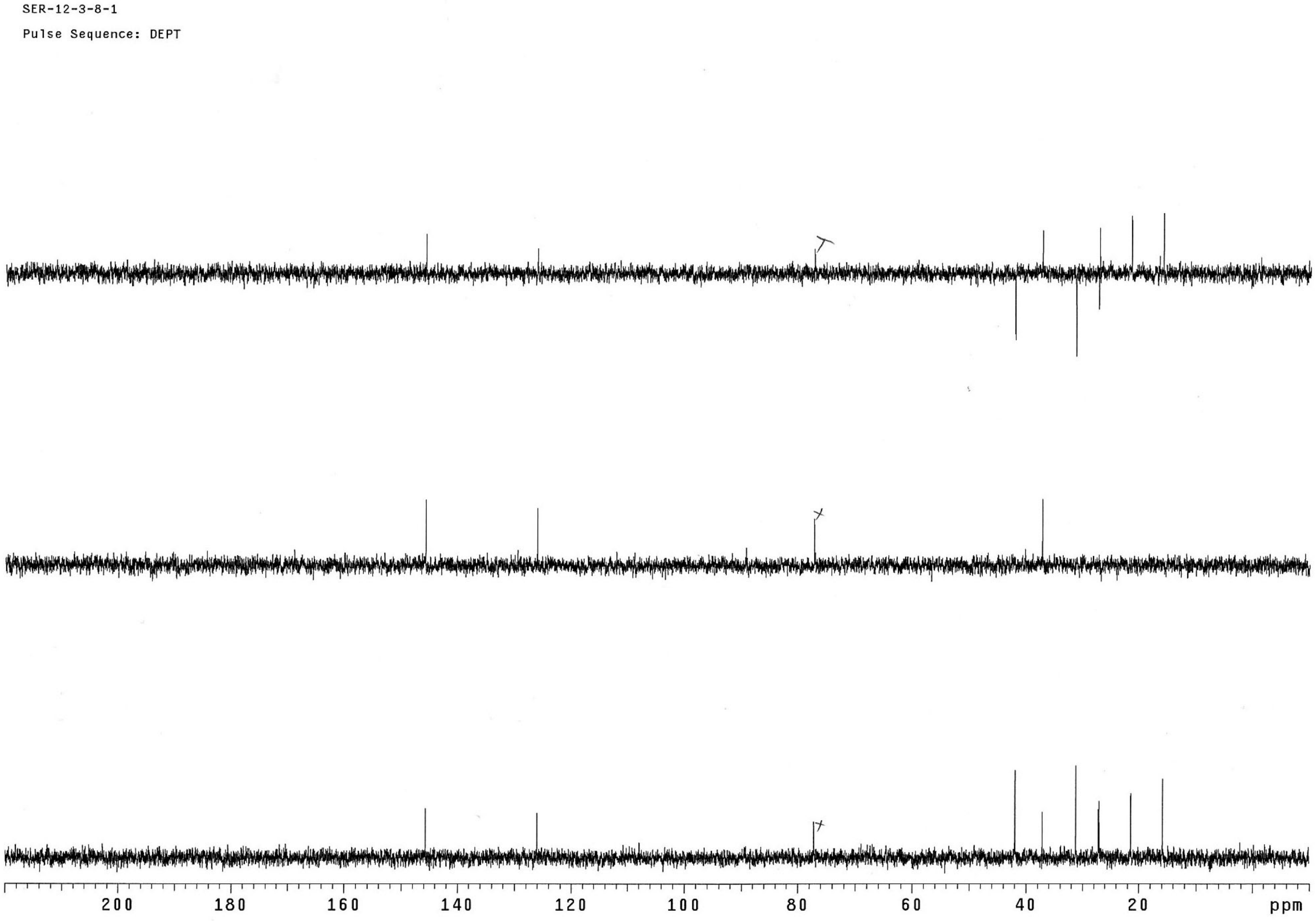

Figure S9. HSQC spectrum of solanerianone A (**1**).

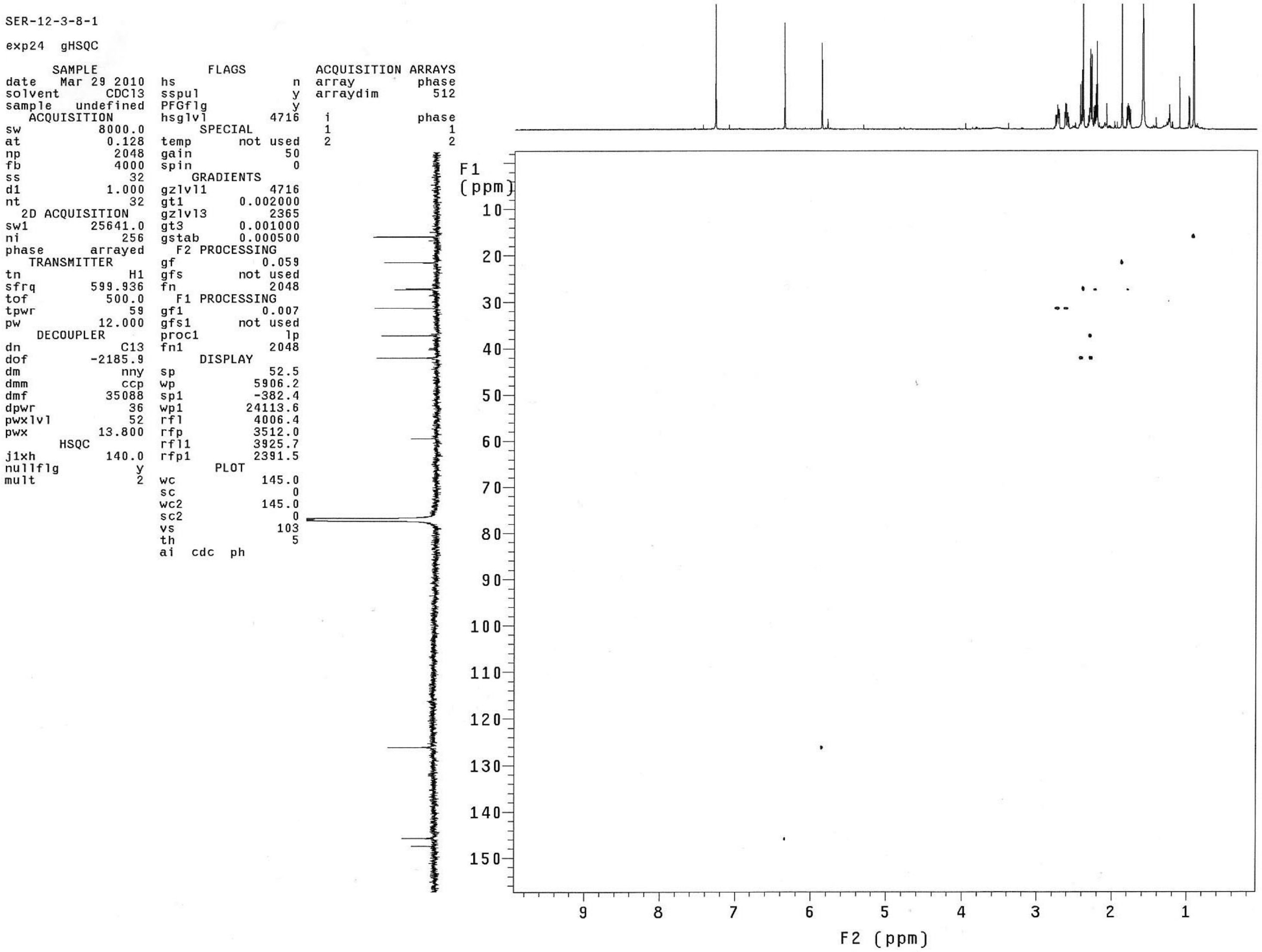

Figure S10. EIMS spectrum of solanerianone B (**2**).

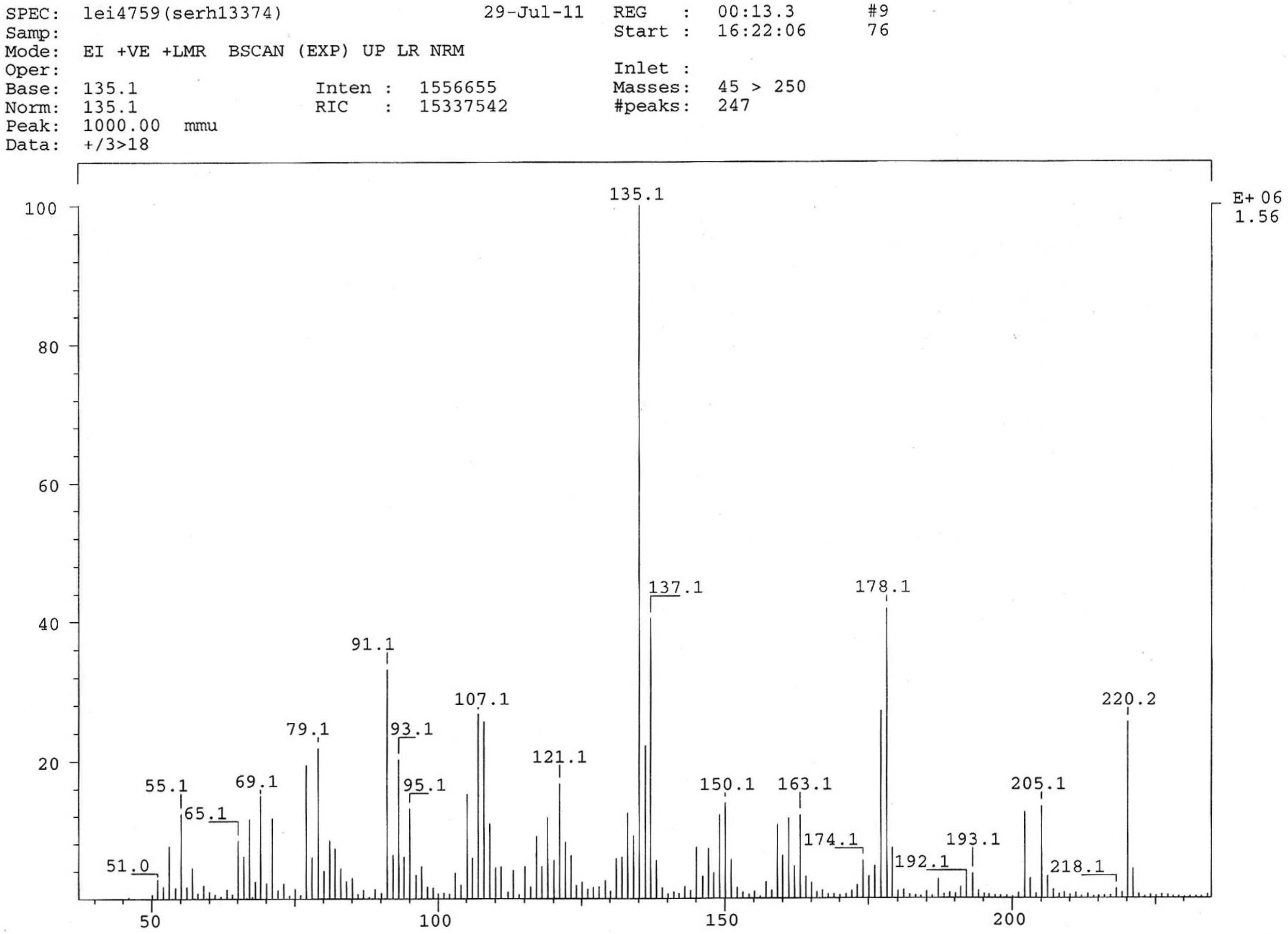

Figure S11. ^{1}H-NMR spectrum of solanerianone B (**2**; 600 MHz, $CDCl_3$).

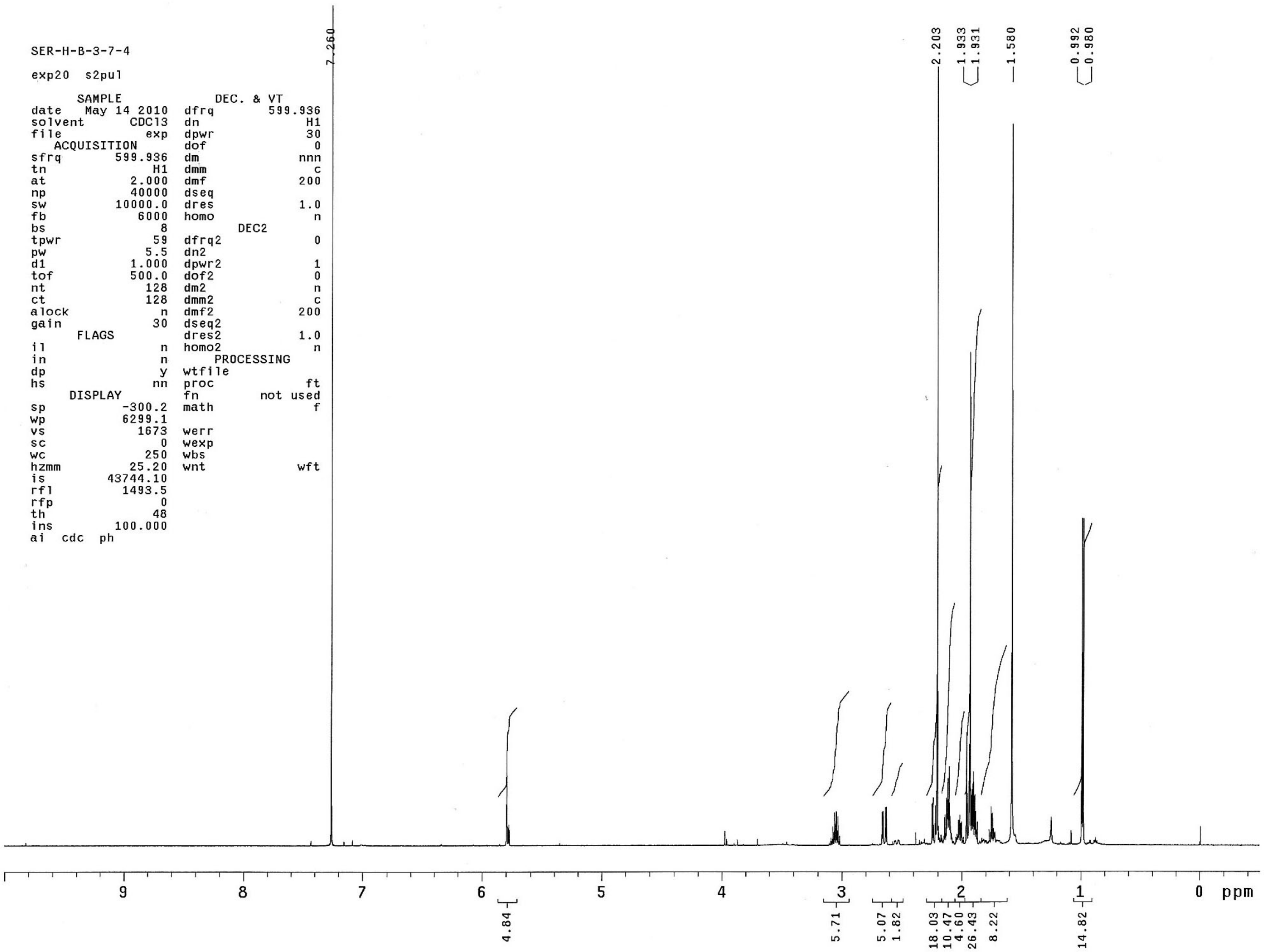

Figure S12. The magnified ^{1}H-NMR spectrum of solanerianone B (**2**; 600 MHz, $CDCl_3$).

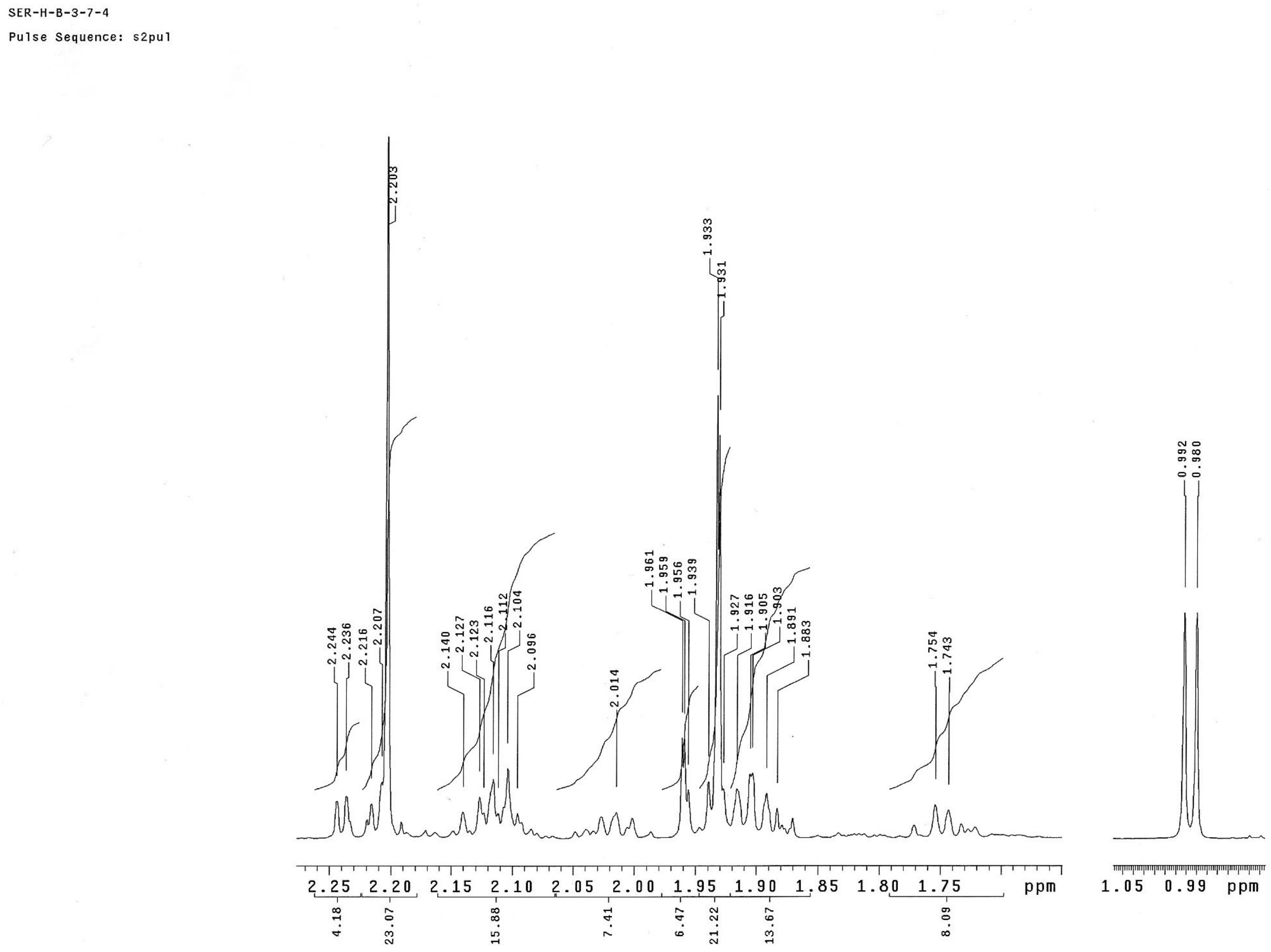

Figure S13. The magnified ^{1}H-NMR spectrum of solanerianone B (**2**; 600 MHz, $CDCl_3$).

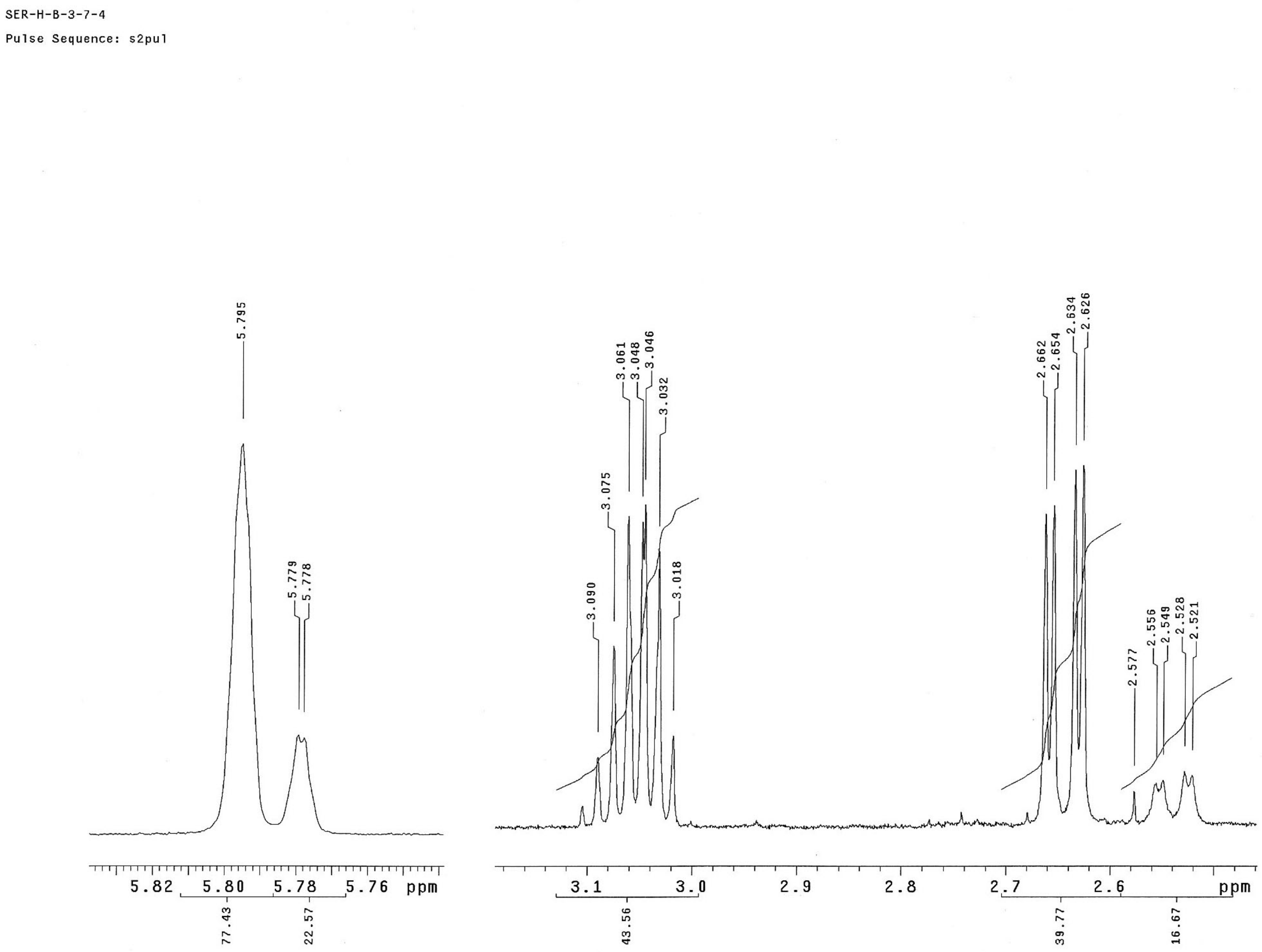

Figure S14. ^{13}C-NMR spectrum of solanerianone B (**2**; 150 MHz, $CDCl_3$).

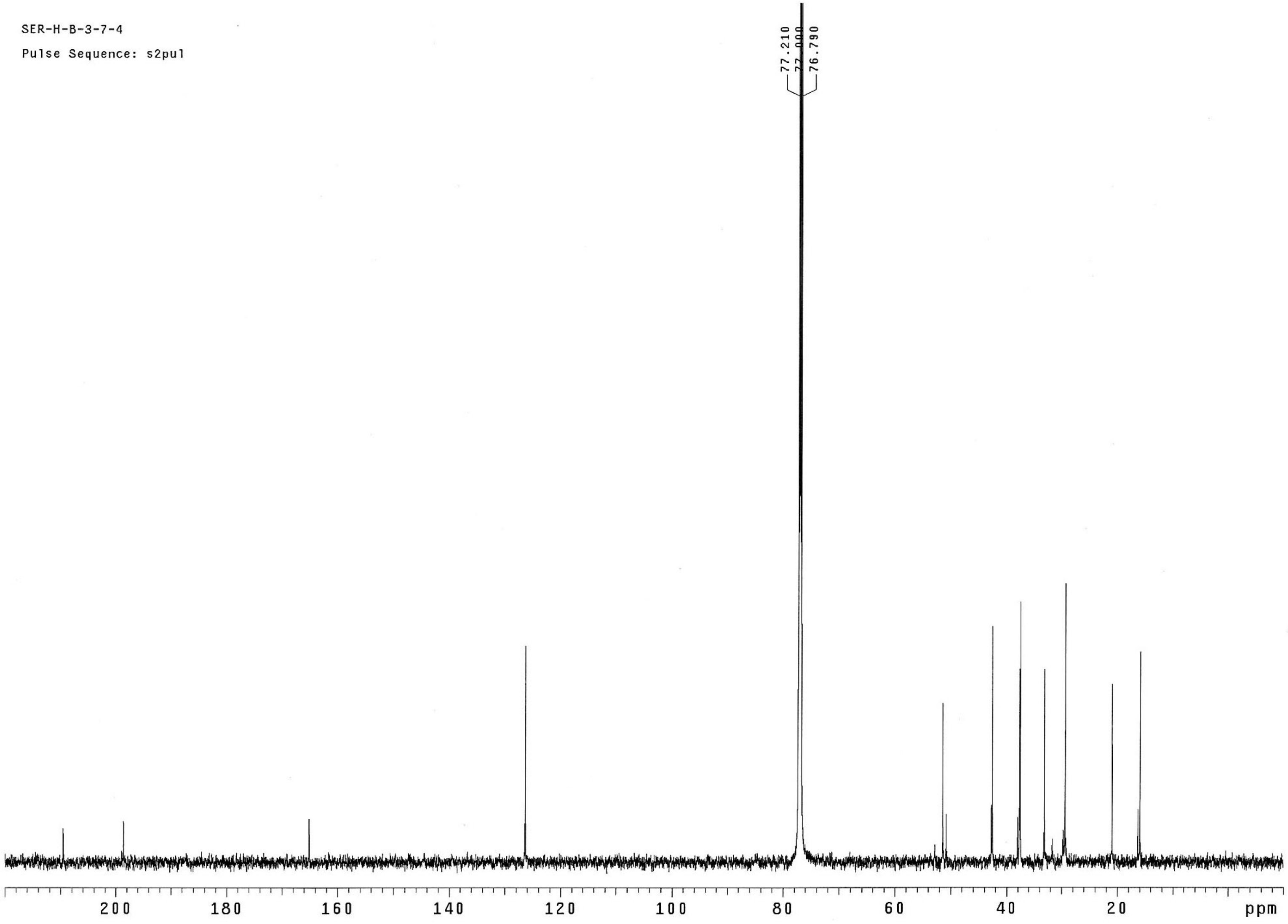

Figure S15. The magnified ^{13}C-NMR spectrum of solanerianone B (**2**; 150 MHz, $CDCl_3$).

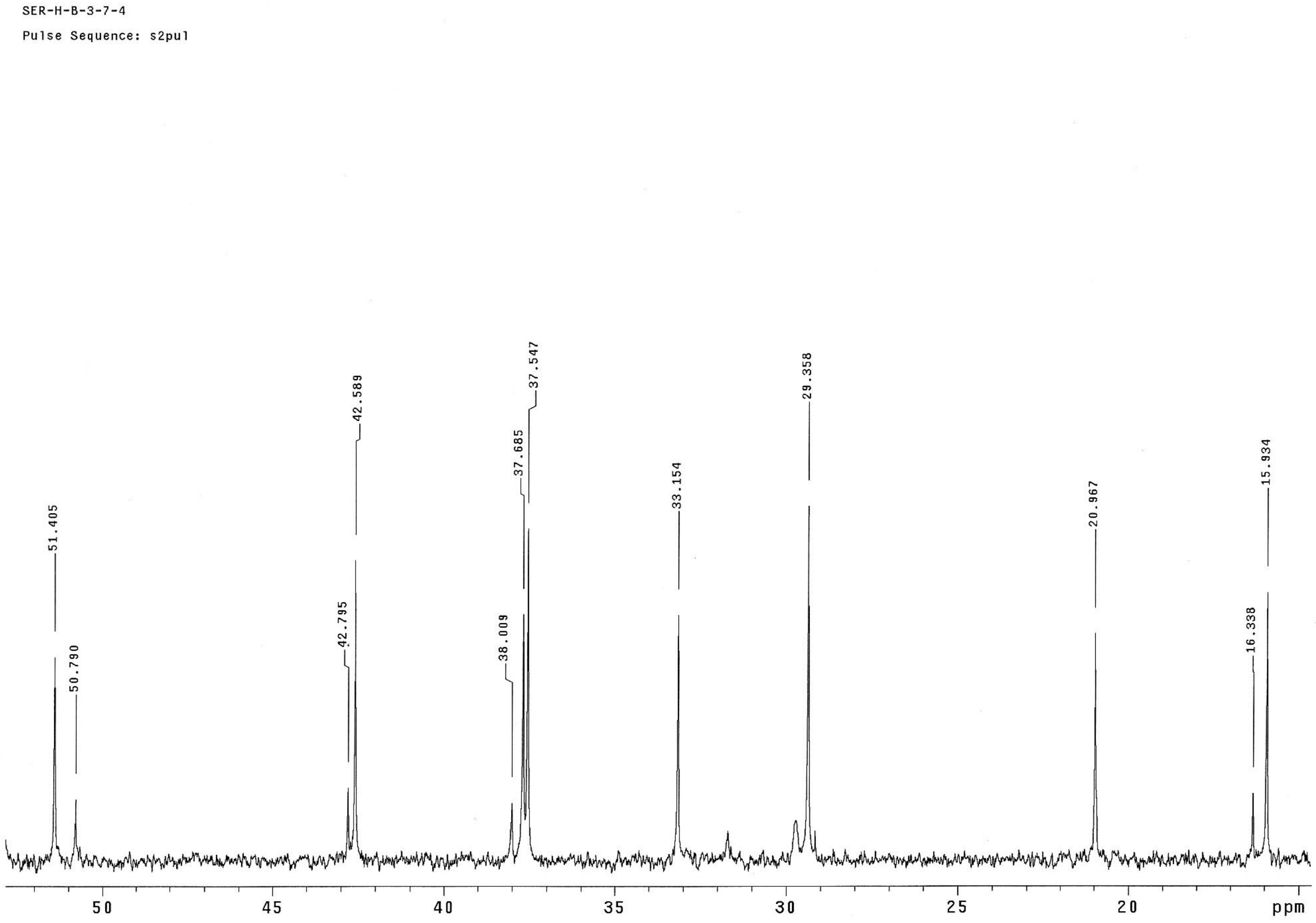

Figure S16. The magnified ^{13}C-NMR spectrum of solanerianone B (**2**; 150 MHz, $CDCl_3$).

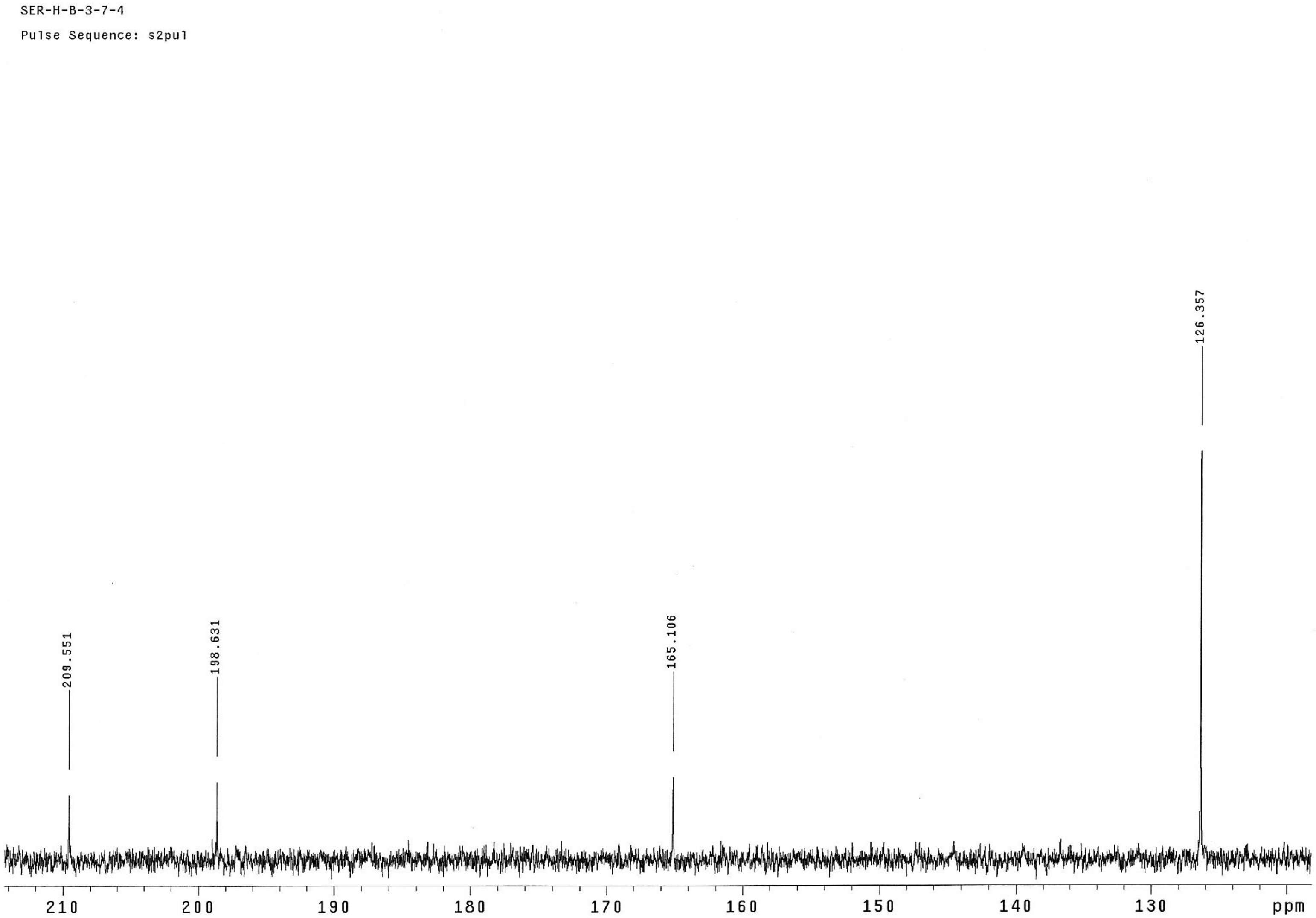

Figure S17. NOESY spectrum of solanerianone B (**2**).

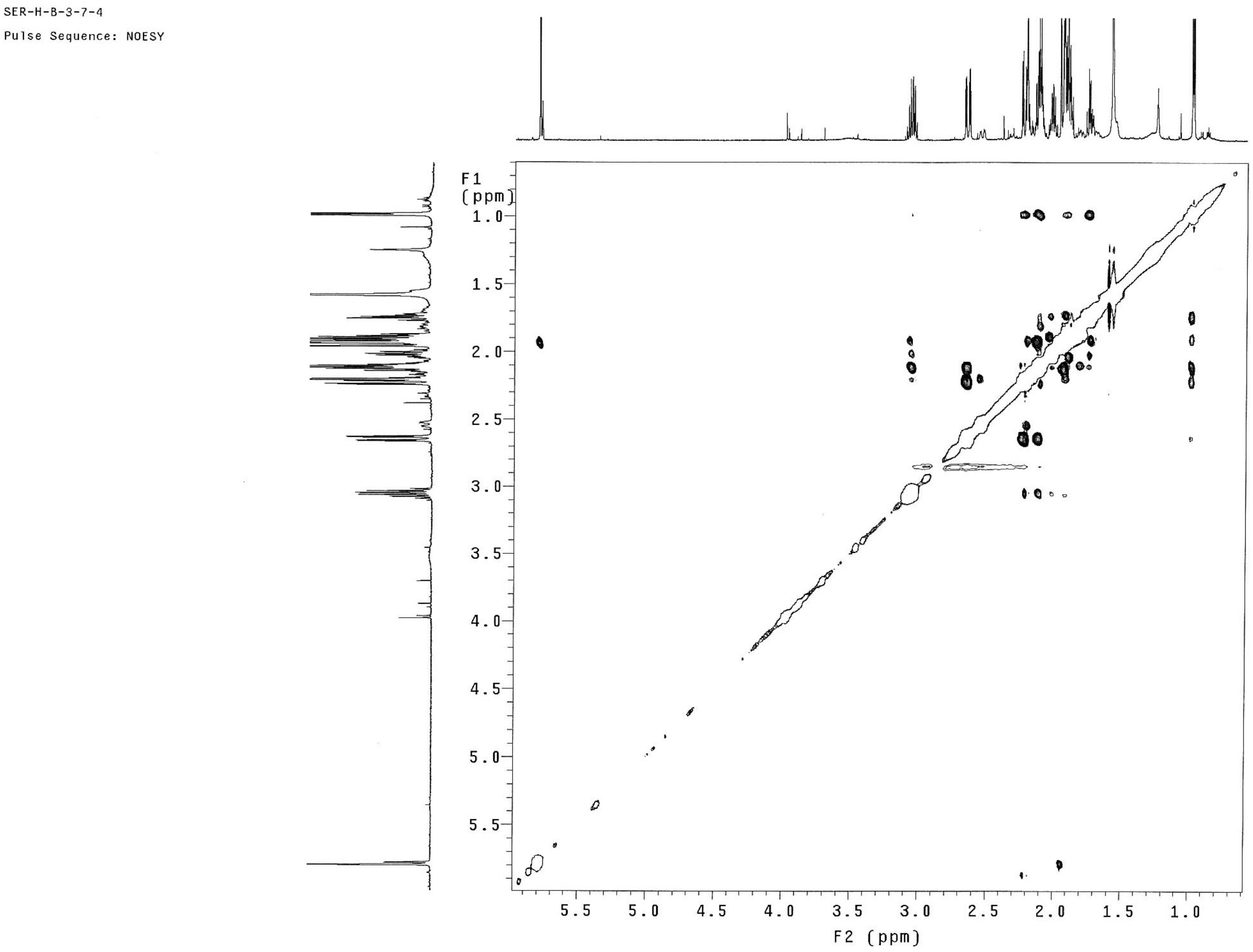

Figure S18. COSY spectrum of solanerianone B (**2**).

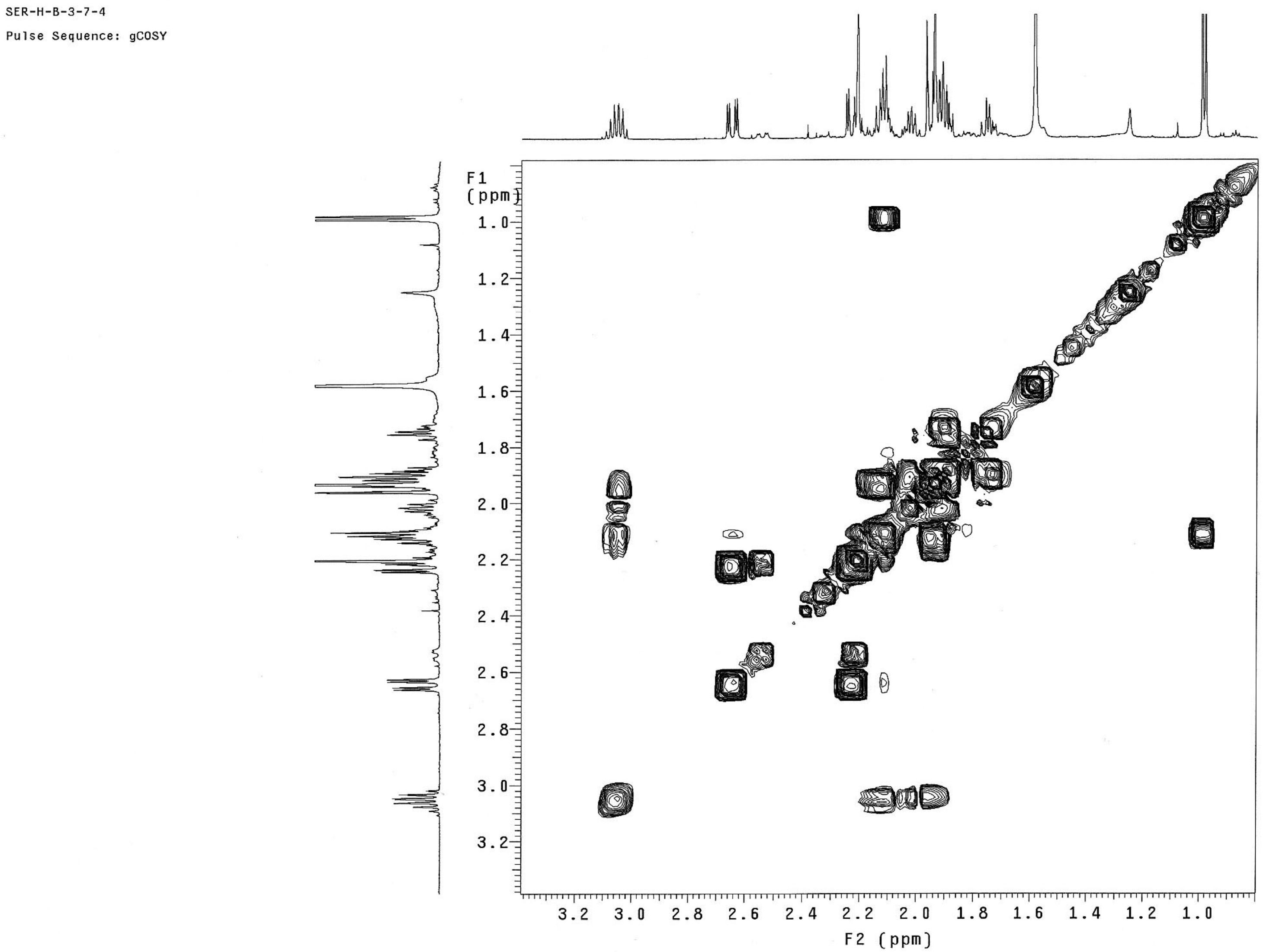

Figure S19. DEPT spectrum of solanerianone B (**2**).

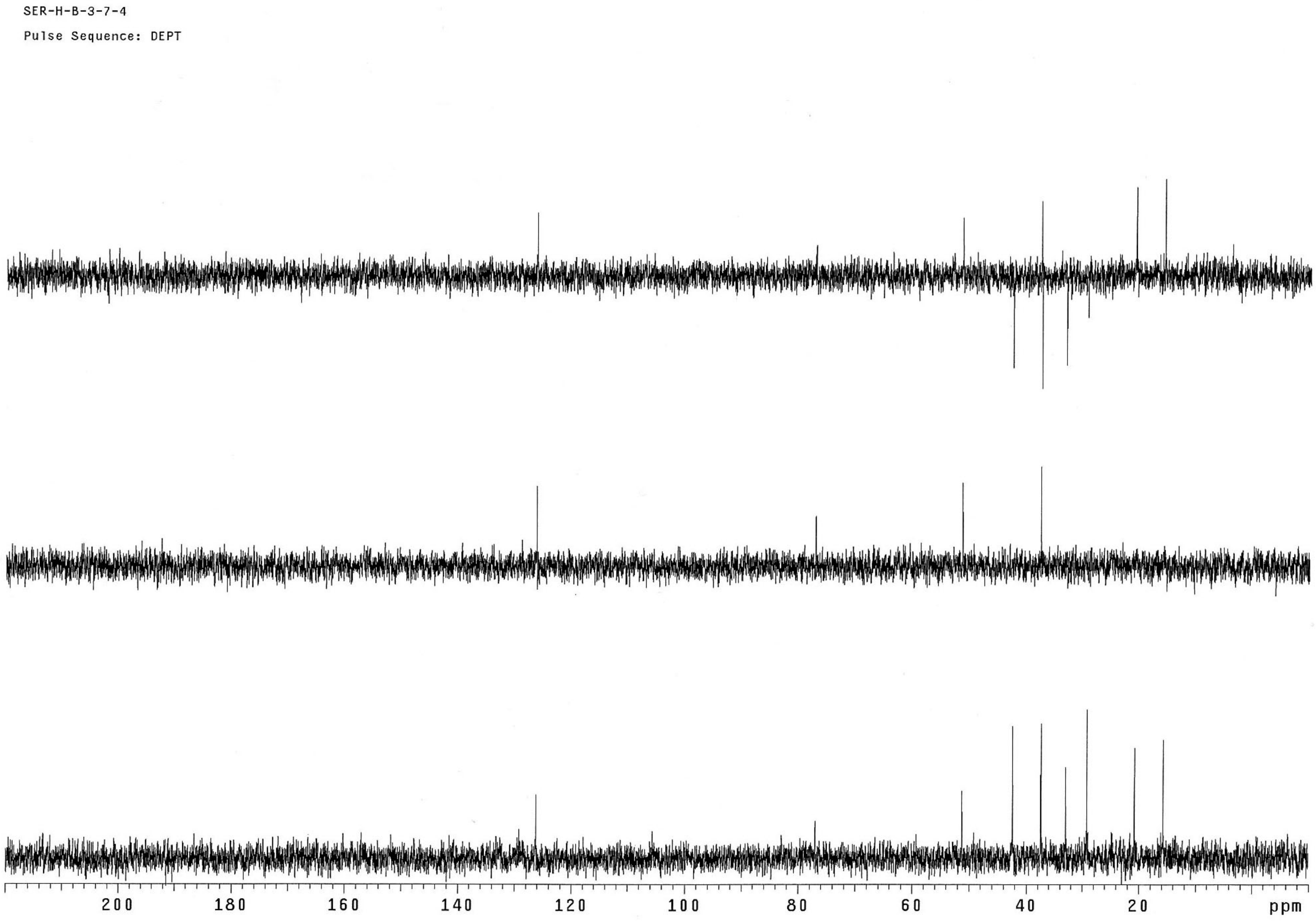

Figure S20. HSQC spectrum of solanerianone B (**2**).

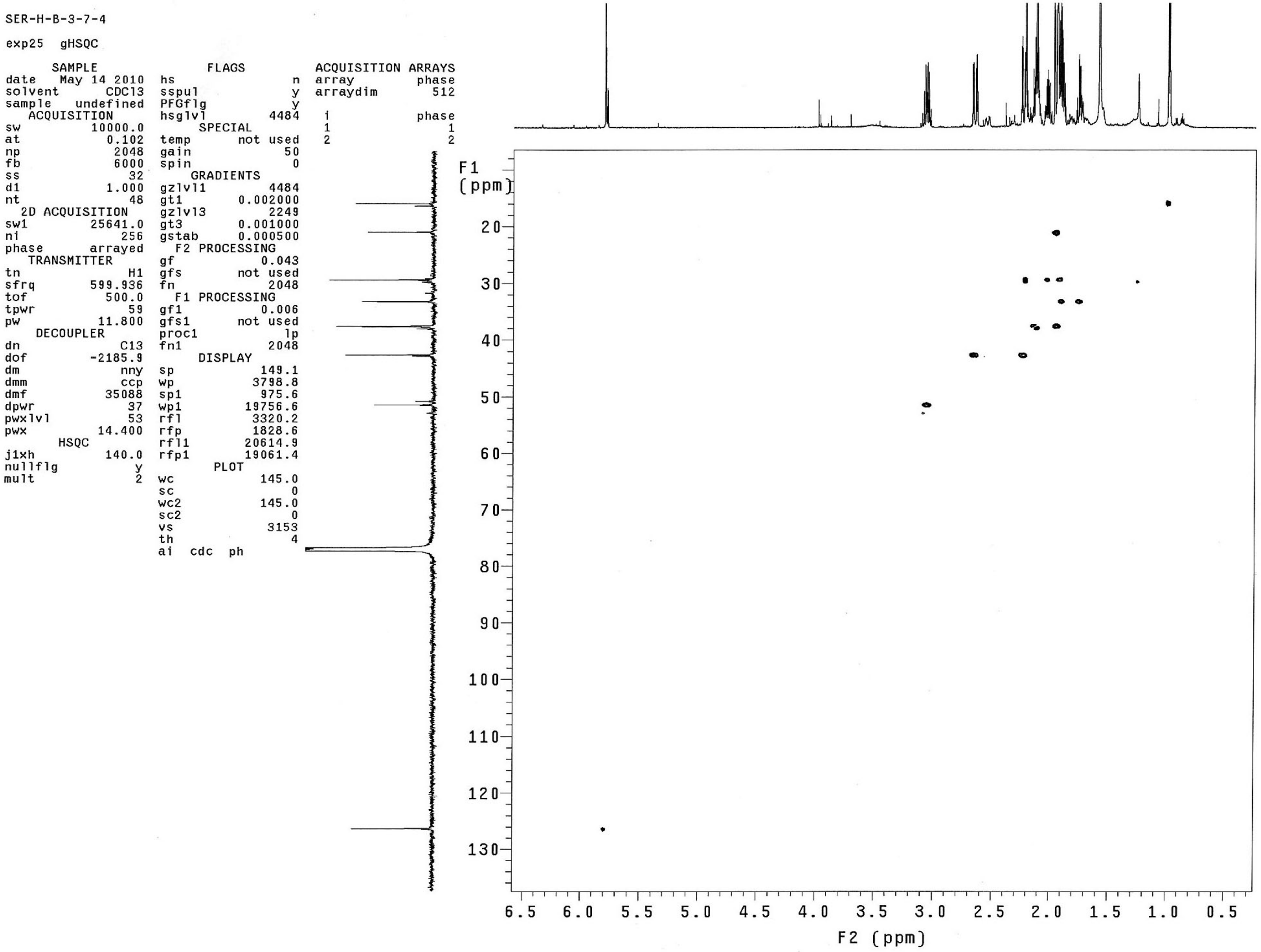

Figure S21. HMBC spectrum of solanerianone B (**2**).

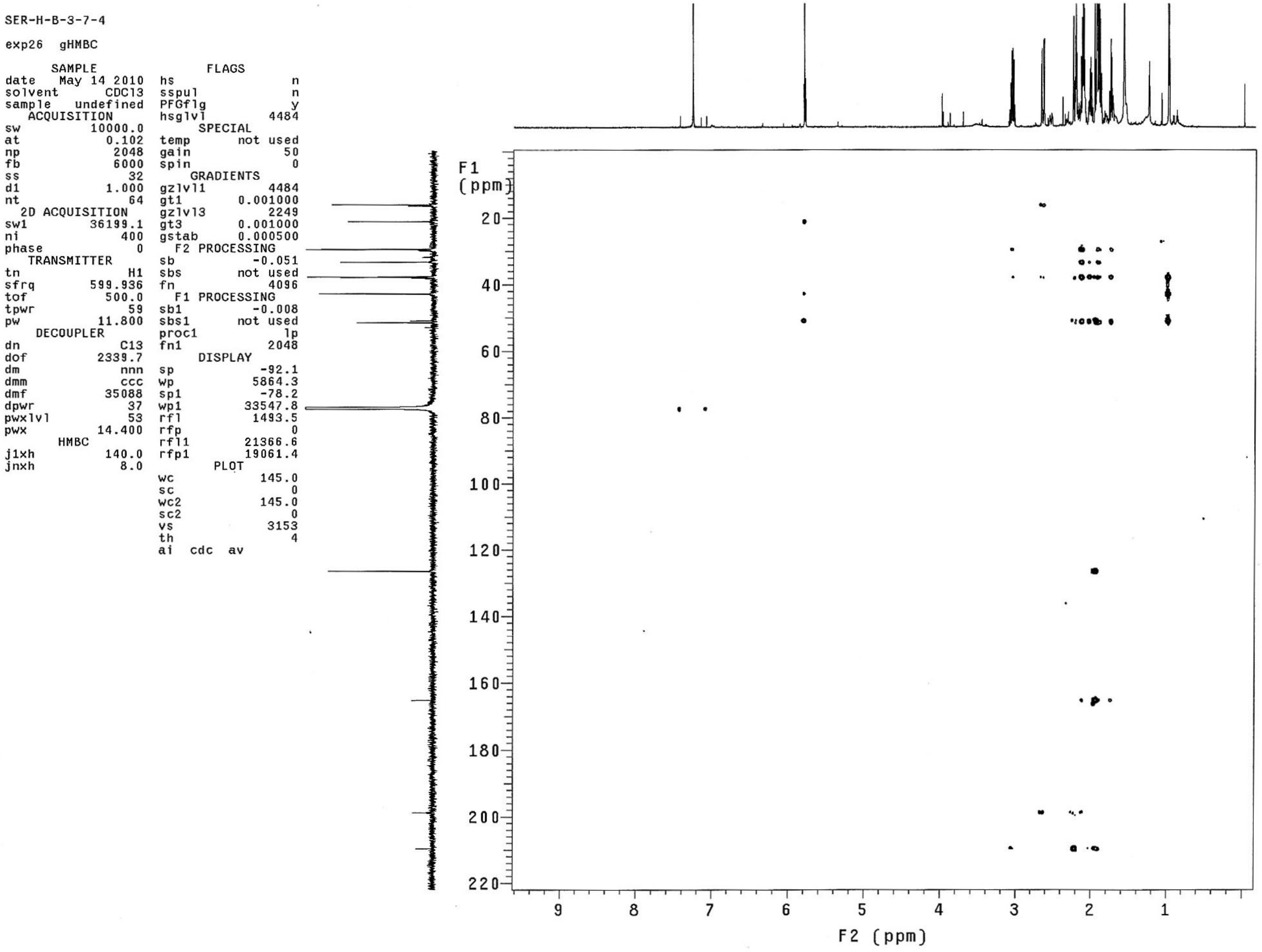

Supplement: Supplementary file 1 [file ijms-14-12581-s001.pdf]
